# Efficacy of phospholipid-bound omega-3 versus standard omega-3 in patients with hypertriglyceridemia: a randomized clinical trial

**DOI:** 10.1186/s12906-026-05245-1

**Published:** 2026-01-10

**Authors:** Miguel Urina-Triana, David Gabriel David-Pardo, Manuel Urina-Triana, Jhesua Valencia, Anthony Bernal-Martínez, Daniela Urina-Jassir, Erick Orozco-Acosta, Tarec K. Elajami

**Affiliations:** 1 Faculty of Health Sciences, Simón Bolívar University, and Fundación del Caribe para la Investigación Biomédica, Barranquilla, Colombia; 2 Fundación del Caribe para la Investigación Biomédica, Barranquilla, Colombia; 3 Division of Cardiology, Hospital Universitario San Ignacio, Pontificia Universidad Javeriana, Bogotá, Colombia; 4 Centro de Investigación en Biotecnología para la vida(CIBIOV), grupo Minciencias, COL 243835, Barranquilla, Colombia; 5 Columbia University Division of Cardiology at Mount Sinai Medical Center, Miami Beach, FL USA

**Keywords:** Bioavailability, EPA, DHA, Hypertriglyceridemia, Omega-3 index, Phospholipid, Randomized controlled trial, Safety

## Abstract

**Background:**

Hypertriglyceridemia is a modifiable risk factor for cardiovascular disease, and while omega-3 fatty acids (omega-3 FAs) are known to lower triglyceride (TG) concentrations, their effectiveness is influenced by formulation and bioavailability. Phospholipid-bound (PL) omega-3 FAs—such as those found in krill oil—demonstrate superior intestinal absorption and membrane integration compared with TG- and ethyl ester omega-3 FAs based forms. Preclinical studies have shown that PL omega-3 FAs exhibit anti-inflammatory, antithrombotic, and TG-lowering properties; however, clinical data remain limited. This pilot randomized clinical trial compared the efficacy of PL omega-3 FAs with standard omega-3 FAs in patients with mild to moderate hypertriglyceridemia.

**Methods:**

We performed a randomized, controlled, double-blind, parallel-group pilot trial involving patients aged 18–65 years with fasting TG between 150 and 499 mg/dL. Patients were randomized to receive PL omega-3 FAs (eicosapentaenoic acid [EPA) + docosahexaenoic acid [DHA), 825 mg/day) or standard omega-3 FAs (EPA + DHA, 903 mg/day). Primary outcomes were changes in fasting TG and omega-3 index (O3I) after 12 weeks. Secondary outcomes included changes in lipid profile, glycemic markers, inflammatory markers, anthropometric measures, hemodynamic variables, and safety parameters.

**Results:**

Of the 47 randomized participants, 44 (22 per group) completed the study and were included in the analysis. Mean TG levels decreased slightly in the PL group (-9.1 mg/dL) and increased in the standard group (+ 15.2 mg/dL), with no statistically significant difference between-groups (*p* = 0.416). Although the O3I increased significantly in both groups, the between-group difference was not statistically significant, despite a greater rise in the PL group (+ 1.78% vs. +1.50%, *p* = 0.446). At week 12, 36.4% of participants in the PL group achieved TG ≤ 150 mg/dL, compared with 13.6% in the standard group (*p* = 0.041). No serious adverse events were reported, and treatment adherence exceeded 95%.

**Conclusions:**

In summary, this pilot randomized trial did not demonstrate statistically significant differences between the PL and TG formulations in triglyceride or Omega-3 Index reduction. However, the numerically higher responder rate and favorable biochemical trends observed with the PL formulation warrant further investigation in larger, adequately powered studies.

**Trial registration:**

NCT06749028 (retrospectively registered on December 19, 2024, after enrollment of the first participant). Available at: https://clinicaltrials.gov/study/NCT06749028.

**European Food Safety Authority (EFSA):**

EFSA-2024-00030369.

**Supplementary Information:**

The online version contains supplementary material available at 10.1186/s12906-026-05245-1.

## Introduction

Hypertriglyceridemia is a common metabolic disorder, affecting approximately 25–30% of the global adult population, and is a well-established risk factor for cardiovascular disease, metabolic syndrome, and pancreatitis [1, 2]. Current therapeutic strategies include lifestyle modifications and pharmacological interventions, among which omega-3 fatty acids (omega-3 FAs), specifically eicosapentaenoic acid (EPA) and docosahexaenoic acid (DHA), are well-established treatments for hypertriglyceridemia. However, their clinical efficacy is influenced by the chemical form, dosage, and bioavailability [3, 4].

Phospholipid-bound (PL) omega-3 FAs, naturally found in krill oil and enriched marine extracts, differ structurally from triglyceride (TG) and ethyl ester forms. In PL omega-3 FAs, EPA and DHA are esterified to a phospholipid glycerol backbone, enabling transport by circulating lipoproteins after intestinal absorption and direct incorporation into the cellular membrane’s phospholipid bilayer. In contrast, TG forms of omega-3 FAs require hydrolysis by pancreatic lipase to release free FAs and monoacylglycerols, which are then re-esterified into phospholipids. This process reduces the efficiency of EPA and DHA integration into cell membranes. Moreover, absorption of TG forms depends on dietary fat intake for optimal uptake, whereas PL forms are not [5–12].

The superior absorption, bioavailability, and cellular membrane incorporation of PL omega-3 FAs have been demonstrated in both preclinical and clinical studies. In a 6-week dietary intervention, krill meal led to a significantly greater increase in the omega-3 index (O3I) compared with fish oil in Alaskan Huskies. Similarly, in humans, PL formulations enhanced the bioavailability of EPA and DHA compared with ethyl ester omega-3 FAs [5]. In an open-label, randomized, four-way crossover trial, including 56 healthy subjects, supplementation with PL omega-3 FAs resulted in 2.7- to 5.0-fold higher plasma concentrations of EPA and DHA compared with the ethyl ester supplement [6].

Beyond systemic bioavailability, PL omega-3 FAs exert a stronger effect on cell membrane structure and function, improving phospholipid bilayer fluidity and modulating cellular signaling, which yields a more potent anti-inflammatory response [7–10]. For example, in an in vivo study of ApoE−/− mice fed a high-fat diet, PL-EPA was associated with lower aortic inflammation, reduced aortic atherosclerotic burden, decreased circulating inflammatory mediators, and improved hepatic and serum lipid profiles [11].

While the antithrombotic effects of omega-3 FAs have been largely attributed to the TG and ethyl ester forms, emerging preclinical evidence suggests that PL omega-3 FAs may also confer antithrombotic benefits. For instance, an in vivo study demonstrated that PL omega-3 FAs delayed platelet-derived thrombin generation and reduced the platelet burden within thrombi [12].

Although emerging preclinical and clinical evidence indicates that PL omega-3 FAs exhibit superior bioavailability and cellular membrane incorporation compared with other forms, clinical data on their pharmacokinetics and biological effects remain limited, underscoring the need for further clinical studies. In the present study, we used a novel PL omega-3 formulation designed to optimize omega-3 FA bioavailability by incorporating EPA and DHA into a matrix of polar phospholipids ( The detailed compositional profile and analytical characterization of both the phospholipid-bound omega-3 formulation and the standard triglyceride fish-oil comparator used in this trial are provided in Supplementary Material 1).

This delivery system is hypothesized to enhance the metabolic efficacy and incorporation of EPA and DHA into the cell membrane. The objective of this trial was to evaluate the biochemical and clinical outcomes of a novel PL omega-3 FA supplement compared with a standard TG omega-3 FA supplement in patients with mild to moderate hypertriglyceridemia.

## Methods

### Study design and participants

This 12-week randomized, controlled, double-blind, parallel-group pilot trial enrolled adults aged 18–65 years with fasting triglyceride levels ≥ 150 mg/dL and < 500 mg/dL.

The 12-week duration was selected based on evidence demonstrating that incorporation of EPA + DHA into erythrocyte membranes and meaningful lipid changes occur within 6–12 weeks.

The inclusion range of fasting triglycerides ≥ 150 mg/dL and < 500 mg/dL was selected because it aligns with international definitions of mild-to-moderate hypertriglyceridemia (AHA/ACC/NCEP guidelines). Individuals within this range represent the population most likely to benefit from dietary and nutraceutical interventions prior to pharmacologic treatment.

Participants were recruited from outpatient cardiovascular prevention clinics in Barranquilla, Colombia. Key exclusion criteria included the use of lipid-lowering medication, uncontrolled diabetes, pregnancy, and omega-3 supplementation within the previous 3 months. Recruitment took place between December 17, 2024, and February 15, 2025.

The study comprised four visits: a baseline screening and enrollment visit, followed by scheduled assessments at weeks 4, 8, and 12. At each follow-up, investigators assessed safety and adherence through structured interviews and pill counts. Adverse events were recorded, graded by severity (mild, moderate, or severe), and evaluated for possible relation to the intervention. The 12-week follow-up concluded in May 2025, with outcome assessments conducted at baseline and week 12.

This clinical trial followed the CONSORT 2025 guidelines. The completed checklist is included as a supplementary file.

### Ethics approval and consent to participate

The study protocol entitled “Efficacy of Phospholipid-Bound Omega-3 Versus Standard Omega-3 in Patients with Hypertriglyceridemia: A Randomized Clinical Trial” was reviewed and approved by the Institutional Research Ethics Committee of the Fundación del Caribe para la Investigación Biomédica (Fundación BIOS) during an ordinary session held on September 3, 2024 (Meeting Minutes No. CEI BIOS-6, Session 0308; Approval Code: CEI BIOS-000001).

The study was conducted in accordance with the ethical principles of the World Medical Association Declaration of Helsinki – Ethical Principles for Medical Research Involving Human Participants (including all amendments). These principles establish internationally recognized standards for research involving human participants and human data, including respect for individual autonomy, protection of participants’ rights and welfare, minimization of risk, scientific validity, and accountability to independent ethical oversight.

The Ethics Committee classified the study as minimal risk. All participants provided written informed consent prior to enrollment after receiving a detailed explanation of the study objectives, procedures, potential risks and benefits, and their rights as research participants. The study was conducted in compliance with the International Council for Harmonisation Good Clinical Practice (ICH-GCP) guidelines and applicable local regulations.

### Patient and public involvement

Patients and members of the public were not involved in the design, conduct, data analysis, or dissemination of this clinical trial.

### Investigational products and blinding

Two omega-3 FA formulations produced by Naturmega S.A. (Barranquilla, Colombia) were evaluated.

The intervention product, *Ruby-O*^®^ Balance, is a phospholipid-bound omega-3 formulation containing a 40/32 EPA/DHA balance and provides a minimum of 400 mg/g of polar lipids (phospholipids and glycolipids). Each 1000 mg capsule delivers approximately 167 mg/g of EPA and 108 mg/g of DHA, both expressed as triglycerides. This formulation was designed to potentially enhance bioavailability and cellular uptake compared to standard triglyceride forms.

The comparator, *Essentiomega™*, is a conventional omega-3 fish oil (TG 18/12) containing 180 mg/g of EPA and 121 mg/g of DHA, as determined by European Pharmacopoeia methods. Both products were manufactured in Colombia under standardized analytical specifications and tested for quality and composition before inclusion in the trial.

Although both commercial products report EPA and DHA values ‘as triglycerides’ in accordance with pharmacopeial and GOED/FDA labeling conventions, this wording reflects a quantitative reporting standard rather than the actual chemical form. In the Ruby-O^®^ Balance formulation, EPA and DHA are predominantly esterified to phospholipids and glycolipids within the polar lipid fraction, not to a triglyceride backbone. This clarification is essential for accurate interpretation of the structural differences between the two investigational products.

A comprehensive description of the analytical methods (column chromatography, GC-FID fatty-acid profiling, and 31P- and ¹H-NMR spectroscopy) used to characterize both investigational products, together with full quantitative composition tables for fatty acids, phospholipid classes, and total EPA + DHA content, is presented in Supplementary Material 1, where the structural and compositional differences between the phospholipid-bound omega-3 formulation and the standard triglyceride fish-oil product are documented in detail.

To maintain blinding integrity, both formulations were manufactured to be identical in appearance, size, color, packaging, and labeling. Naturmega S.A. handled capsule preparation, packaging and labeling to ensure proper masking. Participants and investigators remained blinded to allocation throughout the study.

Both study groups received standardized instructions on dosage and capsule intake, and adherence was monitored at each visit through capsule counts and structured interviews These measures ensured consistent exposure to the assigned omega-3 formulations and minimized potential biases related to adherence and outcome assessment.

### Randomization and treatment allocation

Participants were randomized in a 1:1 ratio to receive either the phospholipid-bound or the standard TG omeda-3 formulation. The randomization sequence was computer-generated using the Sealed Envelope online tool (www.sealedenvelope.com) [13]. Allocation concealment was maintained by an independent pharmacist not otherwise involved in the study.

Blinding was rigorously upheld. Capsules were indistinguishable in appearance—matched in size, color, and packaging—and labeled solely by coded identifiers. Neither participants nor study personnel involved in data collection or outcome assessment had access to group assignments, thereby preserving the double-blind design and minimizing selection bias.

Both groups were instructed to take three capsules daily. The intervention group received *Ruby-O Balance*^®^, providing 825 mg/day of EPA (167 mg/g) and 108 mg/day of DHA (108 mg/g), along with 1,200 mg/day of phospholipids (400 mg/g). The control group received standard fish oil (TG 18/12), delivering 903 mg/day of EPA (180 mg/g) and 121 mg/day of DHA (121 mg/g).

Ruby-O^®^ Balance contains EPA and DHA predominantly bound to phospholipids and glycolipids (≥ 400 mg/g total polar lipids). The comparator formulation (Essentiomega™) provides EPA and DHA exclusively in triglyceride form. The expression ‘EPA and DHA expressed as triglycerides’ reflects the European Pharmacopoeia quantification convention and does not denote the chemical form present in the investigational product. The dose (825 mg/day vs 903 mg/day) was chosen to ensure comparable total EPA + DHA exposure while isolating the effect of the phospholipid vs triglyceride carrier.

Adherence was monitored via capsule counts and participant self-reports at weeks 4, 8, and 12.

### Outcomes and data collection

#### Primary outcomes

The primary outcomes were the change in fasting serum triglycerides (from baseline [TG1] to week 12 [TG4]) and in the O3I over the same period.

#### Secondary outcomes

Secondary endpoints, measured at baseline and week 12, included:

- Lipid profile: high-density lipoprotein cholesterol (HDL-C), low-density lipoprotein cholesterol (LDL-C), total cholesterol (TC), and non-high-density lipoprotein cholesterol (non-HDL-C).
- Glycemic and insulin resistance parameters: fasting glucose, insulin, homeostasis model assessment of insulin resistance (HOMA-IR), triglyceride-glucose (TyG) index, and the TG/HDL-C ratio.
- Inflammatory markers: interleukin-6 (IL-6) and high-sensitivity C-reactive protein (hsCRP).
- Anthropometric measures: body mass index (BMI), waist circumference (WC), hip circumference (HC), and the waist-to-hip ratio (WHR).
- Hemodynamic variables: systolic, diastolic, and mean arterial pressure.

Safety outcomes included hematologic (hemoglobin, hematocrit, leukocytes, platelets, prothrombin time [PT], partial thromboplastin time [PTT], international normalized ratio [INR]), renal (creatinine, blood urea nitrogen [BUN], glomerular filtration rate [GFR) by the CKD-EPI equation), and hepatic parameters (aspartate aminotransferase [AST], alanine aminotransferase [ALT], alkaline phosphatase [ALP], gamma-glutamyl transferase [GGT]), all assessed at baseline and week 12.

Clinical responders were defined as participants meeting at least one of the following criteria: (1) TG4 < TG1; (2) TG4 ≤ 166 mg/dL; (3) TG4 ≤ 156 mg/dL; and (4) TG4 ≤ 150 mg/dL.

#### Lifestyle assessment: physical activity and diet

Physical activity and dietary habits were assessed at baseline and week 12 using structured self-reported questionnaires,

Physical activity was categorized into five levels based on frequency of moderate-to-vigorous exercise: (1) none, (2) occasional (< 2 times/week), (3) moderate (2–3 times/week), (4) regular (4–5 times/week), and (5) daily. Changes were analyzed as ordinal shifts.

Dietary behavior was evaluated using a simplified adherence score based on national dietary guidelines, considering frequency of consumption of key food groups (fruits, vegetables, saturated fats, and sugar-sweetened beverages) and self-perceived adherence to a heart-healthy diet on a 5-point Likert scale.

Both variables were analyzed descriptively to identify potential behavioral trends over time and between groups. These measures were not used for randomization stratification but were considered exploratory outcomes aimed at generating hypotheses regarding lifestyle–treatment interactions.

#### Assessment of adverse events and adherence

Adverse events and adherence were systematically assessed at weeks 4, 8, and 12. At each visit, participants completed structured interviews to identify the occurrence, frequency, and severity of any adverse symptoms experienced. Events were categorized by system, graded by severity, and evaluated for their relationship to the intervention.

Adherence was determined by capsule counts and participant questionnaires. Returned medication containers were inspected at each visit, and the number of unused capsules recorded. Participants also completed a brief adherence questionnaire, reporting missed doses and perceived barriers to compliance. Adherence was considered satisfactory if at least 80% of the prescribed capsules had been consumed between visits.

#### Sample size Estimation

A priori sample size estimation was performed using GPower version 3.1, applying power analysis based on a mixed-model design (repeated measures ANOVA with a within–between interaction). This approach accounted for both intergroup differences and within-subject longitudinal changes over time. The analysis was based on the following assumptions, informed by effect sizes and variability reported in prior omega-3 intervention studies: a moderate expected effect size (f = 0.25, according to Cohen’s criteria), a significance level (α) of 0.05, and a statistical power (1–β) of 0.95, reflecting a 95% probability of detecting a true group × time interaction. The model included two groups (phospholipid-bound omega-3 formulation and control), three measurement points (baseline, midpoint, and endpoint), a correlation of 0.5 among repeated measures, and a nonsphericity correction factor (ε) of 1.0, assuming sphericity.

Under these parameters, the calculated total sample size was 44 participants (22 per group), achieving an actual power of 95.6%. The model yielded a noncentrality parameter (λ) of 16.5, a critical F-value of 3.1052, and degrees of freedom of 2.0 (numerator) and 84.0 (denominator). This sample size was considered sufficient to detect moderate interaction effects in the primary outcomes, triglyceride levels, and O3I. Full details of the analysis are provided in the accompanying GPower output (Annex 1). To account for potential attrition, a 15% anticipated dropout rate was applied, adjusting the final target sample size to 51 participants to ensure adequate statistical power.

### Statistical analysis

Data were analyzed using a per-protocol approach, including only participants who completed the study. No imputation was performed for missing data.

An initial exploratory analysis assessed the distribution and consistency of clinical variables. Normality was evaluated using the Shapiro–Wilk test and confirmed graphically (histograms, boxplots, Q–Q plots). Outliers were identified using the Tukey method (1.5×IQR) and handled transparently to ensure robust interpretation. Variables with high skewness (e.g., triglycerides, IL-6, hsCRP) were analyzed using non-parametric tests, while parametric tests were used when assumptions were met.

Intragroup changes (baseline vs. week 12) were assessed using paired t-tests or Wilcoxon signed-rank tests, depending on data distribution. Intergroup differences were analyzed with independent t-tests, Mann–Whitney U tests, or two-way repeated-measures ANOVA when applicable.

Categorical outcomes, such as the proportion of responders (e.g., TG ≤ 150 mg/dL), were compared using Z-tests for proportions or Fisher’s exact test when expected cell counts were < 5.

To explore variability in treatment response, additional analyses included boxplots, scatterplots of baseline versus final TG and O3I values, and simple linear regressions stratified by treatment group. Response distribution was further examined through threshold analysis, evaluating the proportion of participants exceeding absolute TG reduction cutoffs (≥ 10 to ≥ 150 mg/dL) in each group.

Statistical significance was set at *p* < 0.05 (two-sided). Analyses were performed using SPSS v29 (IBM), R (R Core Team, 2023), and Python. An independent analyst cross-validated all results in compliance with GCP standards.

## Results

**Fig. 1:**
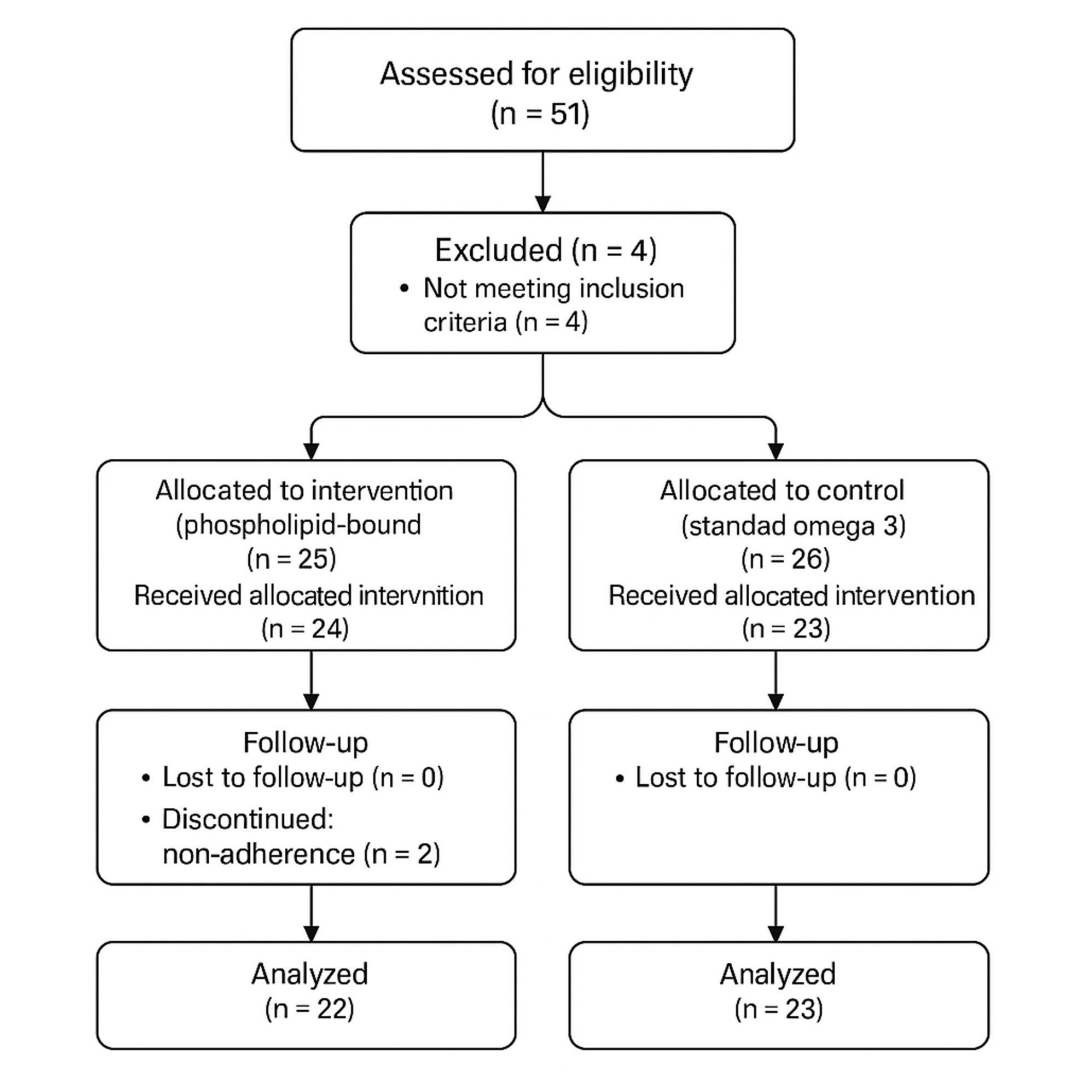
CONSORT flow diagram. Participant flow through the trial, from initial screening (*n* = 51) to final per-protocol analysis (*n* = 44), including reasons for exclusion and discontinuation

A total of 51 individuals were assessed for eligibility. Of these, four were excluded for not meeting the inclusion criteria during the screening phase. The remaining 47 participants were randomly assigned in a 1:1 ratio to receive either the PL-bound omega-3 formulation or standard omega-3 fish oil. During the 12-week follow-up, three participants (two in the intervention group and one in the control group) were excluded from the per-protocol analysis due to treatment non-adherence (< 80% supplement intake). Ultimately, 44 participants (22 per group) completed the study and were included in the final analysis. The participant flow is illustrated in Fig. 1.

### Baseline characteristics

Table 1 summarizes the baseline demographic, clinical, anthropometric, and lifestyle characteristics of the participants by study group. There were no significant between-group differences in terms of age, sex distribution, BMI, systolic or diastolic blood pressure, or waist and hip circumference at baseline. Similarly, baseline TG levels and O3I values were comparable between groups.

Regarding dietary behaviors, most participants reported non-adherence to a healthy diet, with no statistically significant differences between groups in reported intake of added sugars, fruit, or vegetables. Specifically, the majority consumed 1–5 portions of fruit and vegetables per day. Baseline physical activity patterns were comparable between groups, with the highest proportion of participants in both arms reporting no regular exercise, followed by approximately 22.7% engaging in physical activity 2–3 times per week. No significant differences were observed between groups for any baseline characteristic, supporting the adequacy of the randomization process.

**Table 1:** Baseline characteristics of the study population by group. This table presents the demographic and clinical baseline characteristics of participants randomized to receive either the phospholipid-bound omega-3 formulation (intervention group) or the standard omega-3 (control group). Continuous variables are expressed as mean ± standard deviation (SD) or median [interquartile range], as appropriate. Categorical variables are presented as frequency (%). No statistically significant differences were observed between groups at baseline. P-values were calculated using independent t-test, Mann–Whitney U test, or fisher’s exact test, as appropriate

Variable	Control Group (*n* = 22)	Intervention Group (*n* = 22)	*p*-value
Age (years, SD)	44.64 ± 11.37	46.09 ± 11.30	*p* = 0.673
Sex (Female, %)	27.3	36.4	*p* = 0.747
BMI (BMI, SD)	29.30 ± 3.51	30.21 ± 3.99	*p* = 0.425
Systolic BP (mmHg, SD)	124.91 ± 13.34	134.55 ± 25.44	*p* = 0.123
Diastolic BP (mmHg, SD)	81.05 ± 9.44	86.77 ± 13.48	*p* = 0.110
Abdominal Circumference (cms, SD)	100.00 ± 9.24	101.09 ± 8.90	*p* = 0.692
Hip Circumference (cms, SD)	102.36 ± 8.62	105.27 ± 8.42	*p* = 0.264
Triglycerides (mg/Dl, SD)	206.32 ± 72.09	214.10 ± 84.99	*p* = 0.745
Omega-3 Index (%, SD)	5.69 ± 1.12	5.60 ± 1.07	*p* = 0.778
None Diet Adherence (%)	81.8	72.7	*p* = 0.721
Added Sugars Intake (%)	27.3	22.7	*p* = 1.000
Physical Activity			
No exercise (%)	50.0	59.1	*p* = 0.904
Once/week (%)	13.6	9.1	*p* = 0.904
2–3 times/week (%)	22.7	22.7	*p* = 0.904
4–5 times/week (%)	13.6	9.1	*p* = 0.904
Fruit Intake			
None (%)	45.5	40.9	*p* = 1.000
1–5 portions (%)	54.5	59.1	*p* = 1.000
Vegetable Intake			
None (%)	18.2	22.7	*p* = 0.574
1–5 portions (%)	77.3	77.3%	*p* = 0.574
More than 5 portions (%)	4.5%	0.0	*p* = 0.574

### Primary outcomes

#### Triglyceride outcomes and responder analysis

**Fig. 2:**
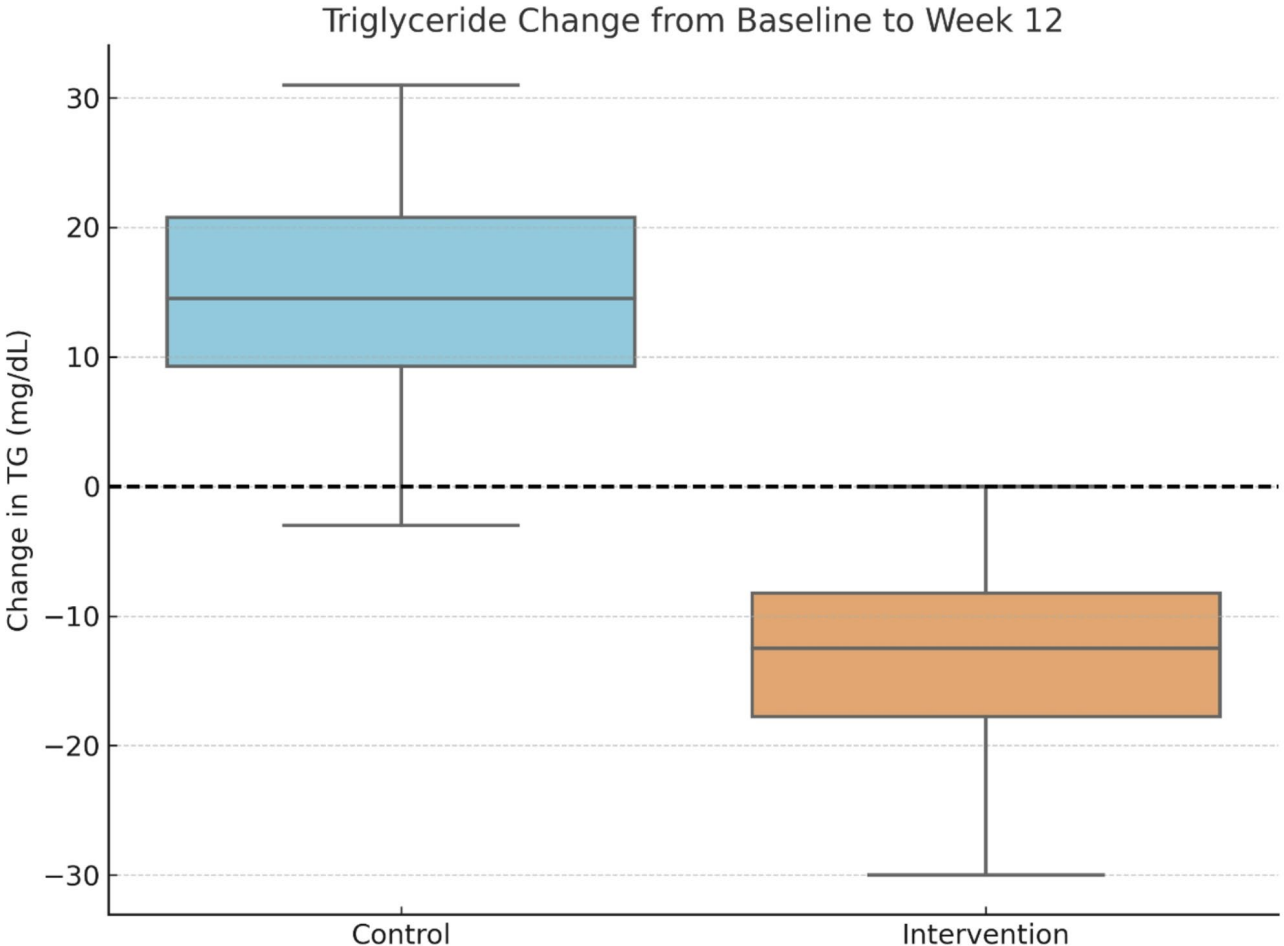
Triglyceride change from baseline to week 12

At baseline, TG levels were comparable between groups. After 12 weeks, participants receiving the PL-bound omega-3 formulation experienced a modest reduction in mean TG levels, from 214.1 ± 85.0 mg/dL to 205.0 ± 93.2 mg/dL, although this change was not statistically significant (*p* = 0.775). In contrast, the control group showed a non-significant increase from 206.3 ± 72.1 to 221.5 ± 75.8 mg/dL (*p* = 0.424). The between-group difference in TG change was also not statistically significant (*p* = 0.416) (Fig. 2).

Boxplot showing change in serum triglyceride (TG) levels in the control group (standard omega-3, *n* = 22) and intervention group (phospholipids bound omega-3, *n* = 22). Median reduction was greater in the intervention group, but the between-group difference was not statistically significant (Mann–Whitney U test, *p* = 0.405).

These results suggest that while the phospholipid-bound omega-3 may have attenuated TG elevation, no significant intra- or inter-group effects were observed.

**Fig. 3:**
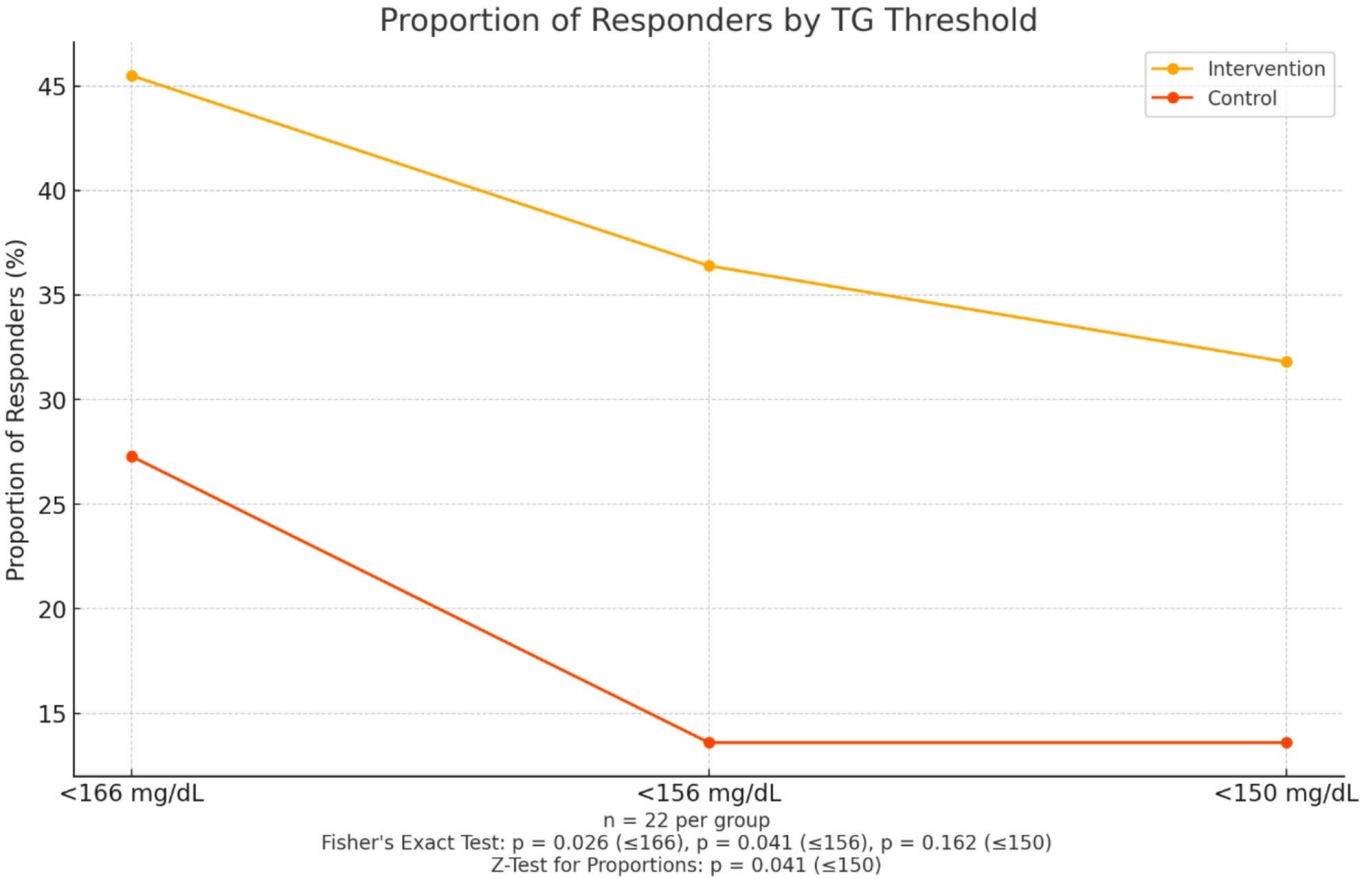
Triglyceride responder rates by clinical thresholds

To better understand the clinical relevance of these findings, a responder analysis was conducted (Fig. 3). A significantly higher proportion of patients in the PL omega-3 formulation group achieved a reduction in TG from baseline to week 12 (63.6%, 14/22), compared with the control group (36.4%, 8/22; *p* = 0.035).

Furthermore, a threshold-based analysis evaluated the proportion of participants achieving TG levels below predefined clinical targets. At the ≤ 166 mg/dL cutoff, 50.0% (11/22) of participants in the PL omega-3 group met the target, versus 18.2% (4/22) in the control group (*p* = 0.026; RR = 2.75). At the ≤ 156 mg/dL cutoff, 40.9% (9/22) in the PL omega-3 group met the criterion versus 13.6% (3/22) in the control group (*p* = 0.041; RR = 3.00). At the widely accepted hypertriglyceridemia cutoff (≤ 150 mg/dL), 36.4% (8/22) of PL omega-3 treated participants reached the target, compared to 13.6% (3/22) in the control group. Although Fisher’s exact test did not reach statistical significance at this threshold (OR = 3.62; *p* = 0.162), a one-tailed Z-test for proportions confirmed a statistically significant difference (z = 1.74, *p* = 0.041).

These findings support a potential clinical advantage of the phospholipid-based omega-3 formulation in achieving evidence-based TG targets and reinforce its potential role as an adjunctive therapeutic strategy for patients with mild to moderate hypertriglyceridemia.

Percentage of participants achieving triglyceride levels below three clinical cut-off points (< 166, < 156, and < 150 mg/dL) after 12 weeks. The intervention group (phospholipid-bound omega-3 = intervention) showed significantly higher responder rates than the control group at < 166 and < 156 mg/dL (Fisher’s Exact Test: *p* = 0.026 and *p* = 0.041, respectively; Z-Test for Proportions: *p* = 0.041 at ≤ 150 mg/dL).

**Fig. 4:**
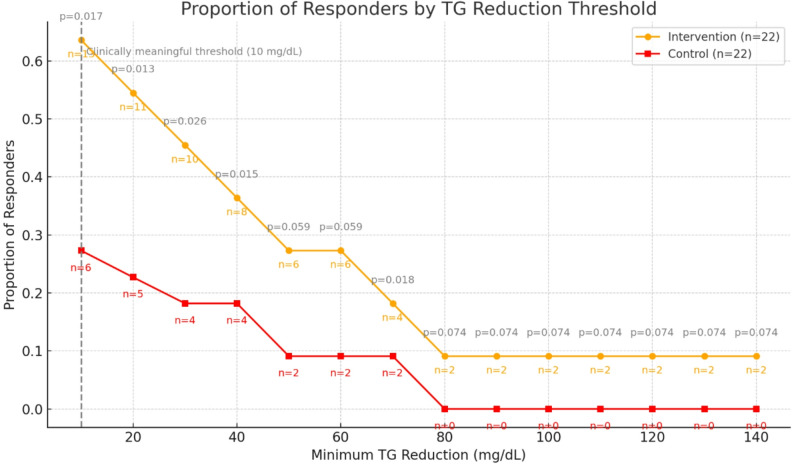
Proportion of responders achieving various thresholds of triglyceride (TG) reduction after 12 weeks of supplementation. The graph depicts the proportion of participants in each group—intervention (phospholipid-bound omega-3; *n* = 22) and control (standard omega-3; *n* = 22)—who achieved reductions in TG of ≥ 20, ≥40, ≥ 60, ≥80, ≥ 100, ≥120, and ≥ 140 mg/dL. The intervention group consistently demonstrated a higher proportion of responders across all thresholds, with a statistically significant difference in overall response rates (*p* = 0.017)

To complement these categorical findings, a responder threshold analysis was also conducted across absolute TG reduction cutoffs ranging from ≥ 10 mg/dL to ≥ 140 mg/dL. The PL omega-3 group consistently showed higher responder rates at each threshold. For instance, 63.6% (*n* = 14) of participants in the phospholipid-bound group achieved at least a 10 mg/dL reduction, compared to 27.3% (*n* = 6) in the control group (*p* = 0.017). This trend remained significant through increasingly stringent cutoffs, with significant differences observed up to the 70 mg/dL threshold. Beyond this point, responder rates declined in both groups, but only participants in the phospholipid-bound group continued to meet higher reduction thresholds (up to 140 mg/dL), while none in the control group did. These results reinforce the superior triglyceride-lowering efficacy of the PL omega-3 formulation and its potential capacity to achieve both moderate and intensive lipid targets (Fig. 4).

**Fig. 5:**
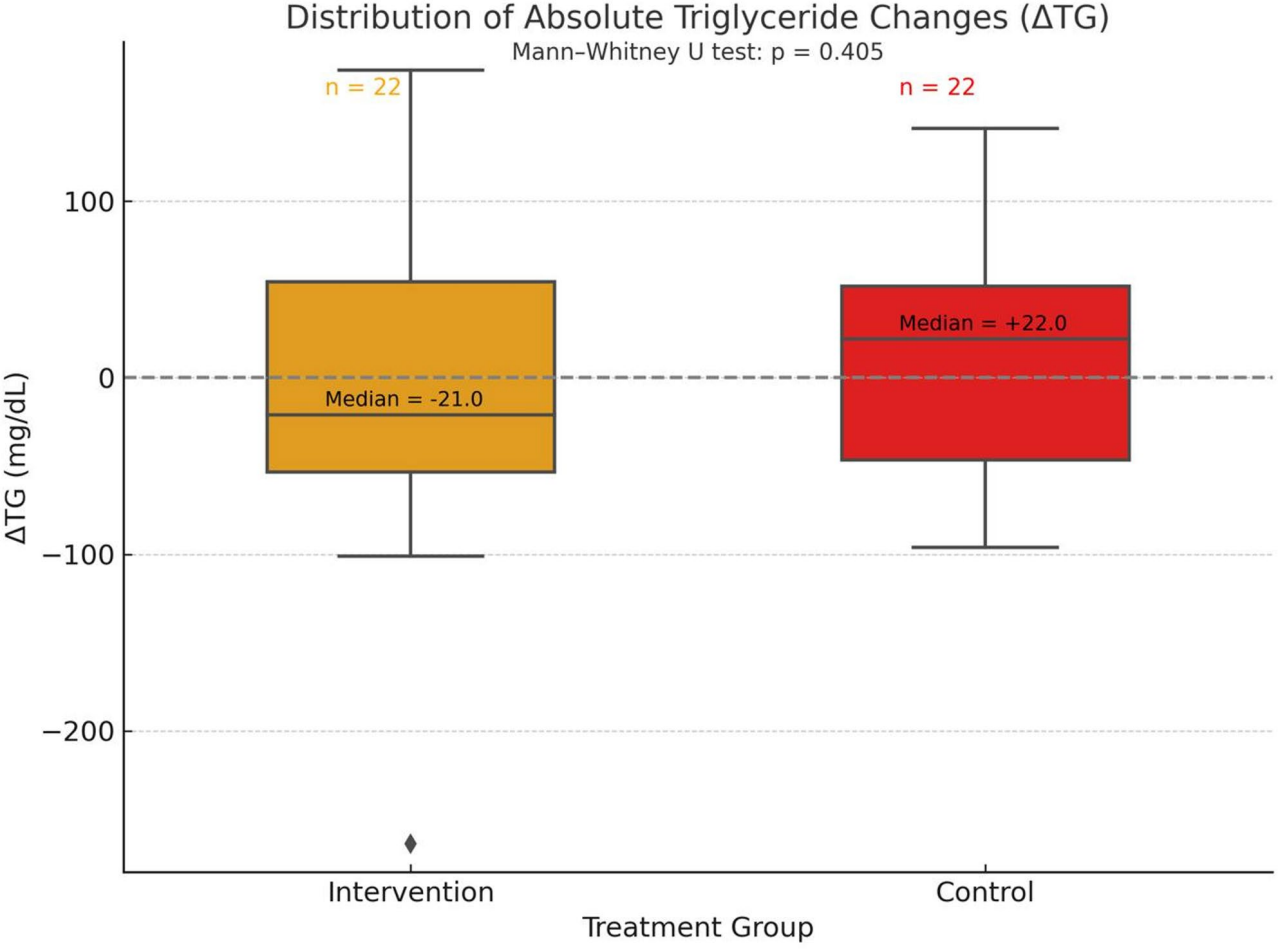
Change in serum triglyceride (TG) levels from baseline to 12 weeks by treatment group

A distributional analysis of absolute triglyceride changes (ΔTG) using boxplots was performed to evaluate inter-individual variability. The median ΔTG was − 21 mg/dL in the phospholipid-bound omega-3 group and + 22 mg/dL in the control group, yielding a median difference of − 43 mg/dL favoring the intervention. Although this difference was not statistically significant (*p* = 0.405, Mann–Whitney U test), the distributional shift supports a consistent trend toward TG reduction among participants receiving the phospholipid-bound formulation (Fig. 5).

Boxplots illustrate the distribution of TG changes (final minus baseline) in both groups: intervention group (phospholipid-bound omega-3 formulation, *n* = 22) and control group (standard omega-3 fish oil, *n* = 22). The median reduction was greater in the intervention group, with a wider interquartile range indicating more variability. Although a trend toward a greater TG decrease was observed in the intervention group, the between-group difference was not statistically significant (*p* = 0.416, Mann–Whitney U test).

Moreover, 63.6% of participants in the phospholipid-bound omega-3 group experienced a net TG decrease, compared to 36.4% in the control group. Notably, TG reductions exceeding 100 mg/dL occurred exclusively in the phospholipid group, highlighting its potential to elicit more pronounced lipid-lowering effects in certain individuals—effects that may otherwise be obscured when relying solely on mean-based comparisons.

This graphical, non-parametric approach is particularly appropriate for skewed clinical variables such as TG, where reporting medians and interquartile ranges (IQRs) provides a more accurate and clinically meaningful summary than means and standard deviations. In line with best practices for non-normally distributed outcomes, results were expressed using the five-number summary (minimum, Q1, median, Q3, maximum).

In summary, these distribution-based findings reinforce the primary efficacy results: PL omega-3 increased the proportion of responders and enabled greater TG reductions in a subset of participants, underscoring its therapeutic potential for managing mild-to-moderate hypertriglyceridemia.

#### Omega-3 index outcomes and Pharmacokinetic efficiency

The O3I, defined as the percentage of EPA and DHA incorporated into erythrocyte membranes, was measured at baseline and after 12 weeks as a biomarker of long-term omega-3 tissue integration. Both groups exhibited statistically significant increases in O3I following supplementation.

**Fig. 6:**
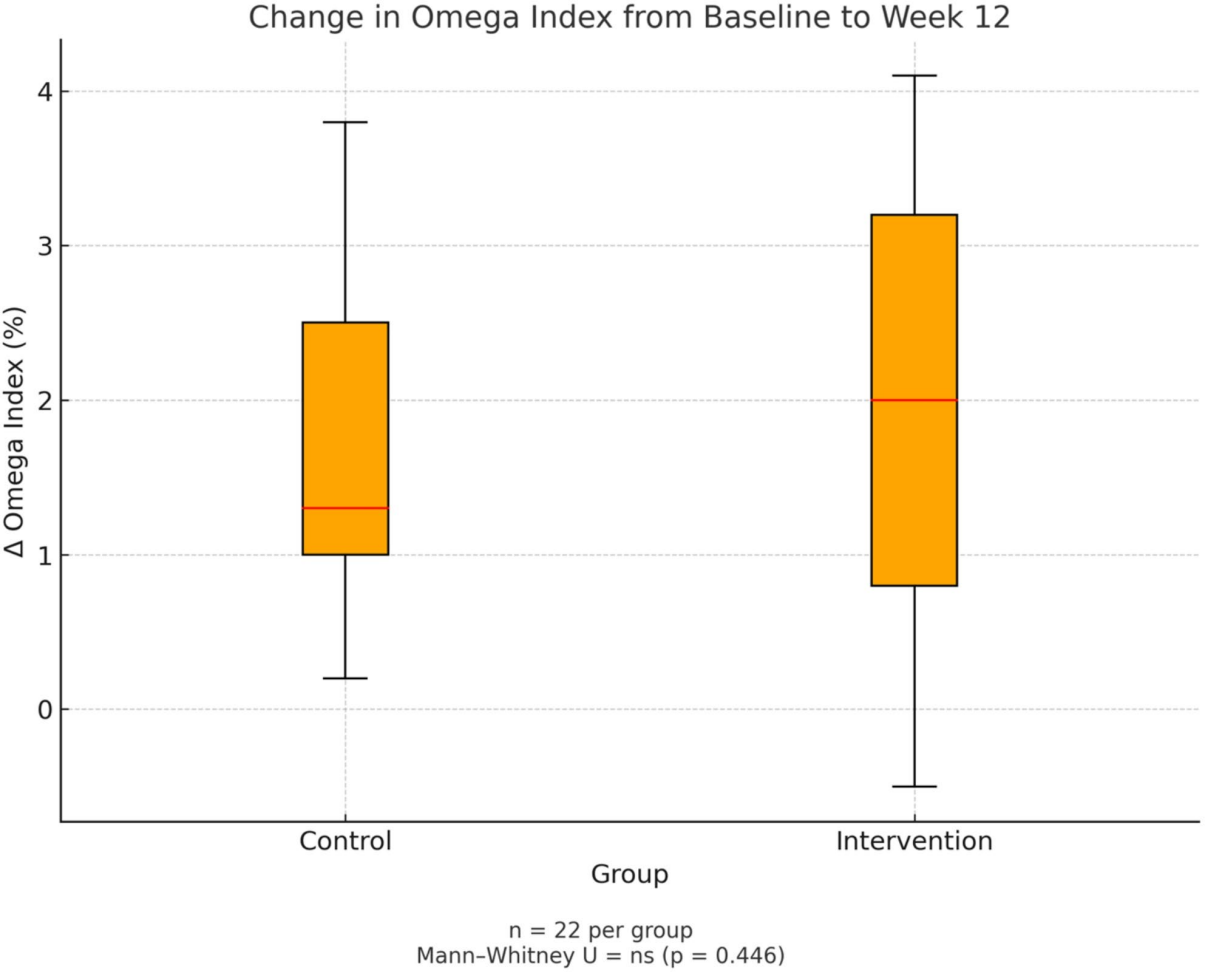
Change in omega-3 Index from baseline to week 12

In the control group, the O3I increased from 5.69 ± 1.12% to 7.19 ± 1.38% (mean change: +1.50 ± 1.19%; *p* < 0.00001), while in the PL omega-3 formulation group it increased from 5.60 ± 1.07% to 7.38 ± 1.44% (mean change: +1.79 ± 1.25%; *p* < 0.000001). Although the PL omega-3 formulation group showed a numerically greater increase, the between-group difference was not statistically significant (*p* = 0.446, Mann–Whitney U test) (Fig. 6).

Boxplot illustrating the change in omega-3 index (%) after 12 weeks of supplementation. Both groups showed a comparable increase, with no statistically significant difference between the intervention group (phospholipid-bound omega-3) and the control group (standard omega-3) (Mann–Whitney U test, *p* = 0.446).

To further assess the pharmacokinetic performance of each formulation, we evaluated the relative percentage increase in O3I per participant. The PL omega-3 formulation group achieved a mean relative increase of 34.15%, compared to 27.95% in the control group. Median relative increases were similar between groups (PL: 27.33%; Control: 27.36%), with no statistically significant difference (*p* = 0.474).

However, after adjusting for total daily EPA + DHA dose (825 mg/day for PL omega-3 formulation versus 903 mg/day for control), the PL omega-3 formulation demonstrated superior dose efficiency. The dose-normalized O3I response was 0.0422% per mg of EPA + DHA for phospholipid-bound omega-3 formulation versus 0.0311% per mg for the control group, yielding a Relative Efficiency Index (REI) of 1.34. This indicates that phospholipid-bound omega-3 formulation achieved 34% greater biological incorporation of omega-3 per milligram of active ingredient compared to conventional fish oil.

This exploratory, post hoc analysis supports the hypothesis that phospholipid-bound omega-3 formulations may enhance gastrointestinal absorption and tissue incorporation of EPA and DHA compared to standard TG-based products.

#### Subgroup regression analysis by treatment group

To further explore differential response patterns, linear regression analyses were performed separately for each treatment arm.

#### Triglyceride outcomes (TG4 vs. TG1)

**Fig. 7:**
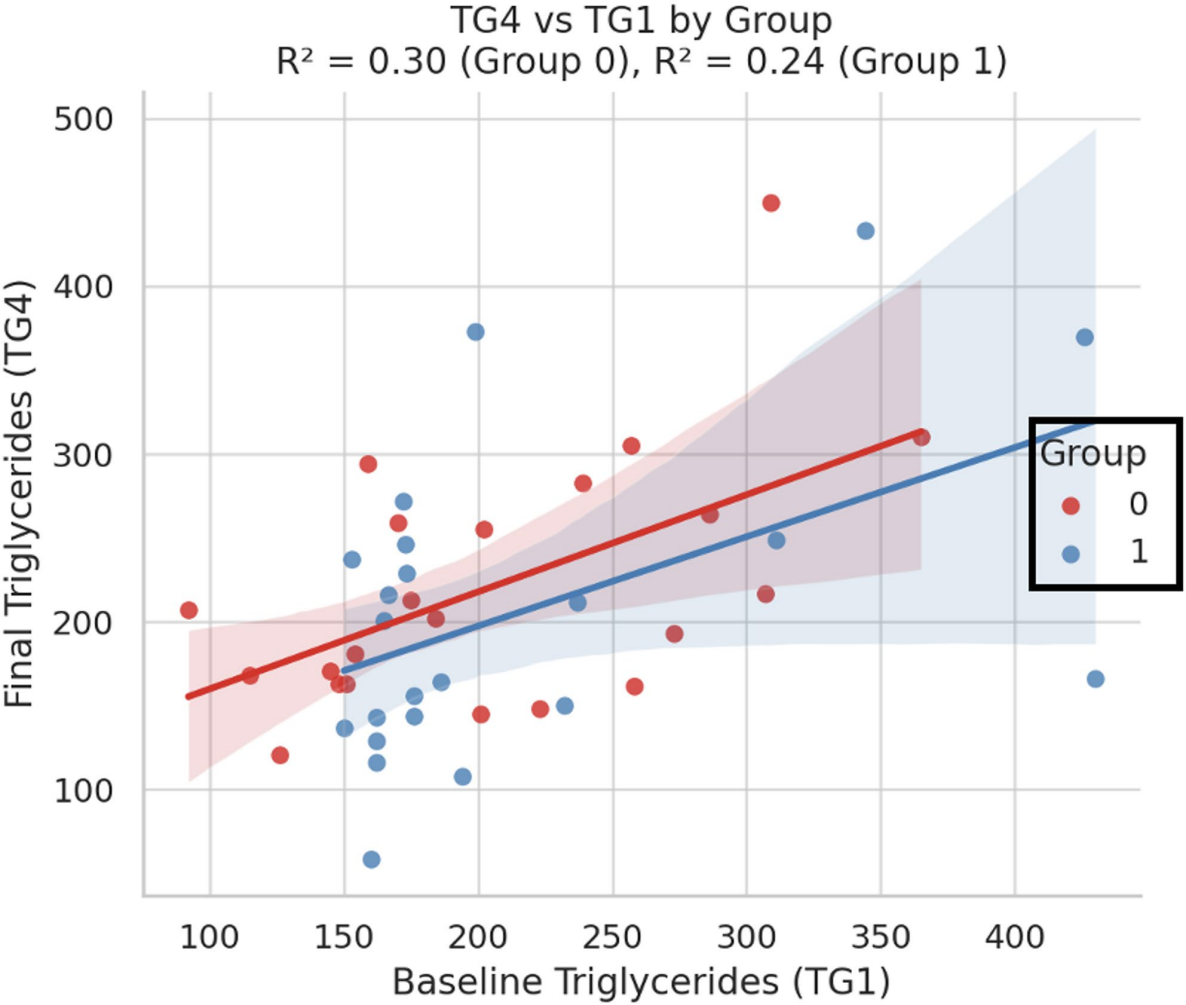
Correlation between baseline and final triglyceride levels by group

The stratified regression plot (Fig. 7) shows a positive association between baseline TG levels (TG1) and final levels (TG4) in both groups. Notably, the control group exhibited a steeper slope (R² = 0.30), suggesting that individuals with higher baseline TG tended to maintain proportionally higher levels after receiving standard omega-3 supplementation. In contrast, the PL omega-3 group showed a flatter slope (R² = 0.24), indicating a potential buffering effect on post-treatment TG levels, especially among participants with elevated baseline TG.

Scatter plot with linear regression lines showing the association between baseline triglycerides (TG1) and final triglycerides (TG4) in both groups. The control group (red line = 0) demonstrated a stronger correlation (R² = 0.30), suggesting that higher initial TG levels predicted higher final values. In contrast, the intervention group receiving phospholipid-bound omega-3 (blue line = 1) exhibited a flatter slope and lower correlation (R² = 0.24), indicating a potential attenuating effect on TG elevation among individuals with higher baseline levels.

#### Omega-3 index outcomes (O3I4 vs. O3I1)

**Fig. 8:**
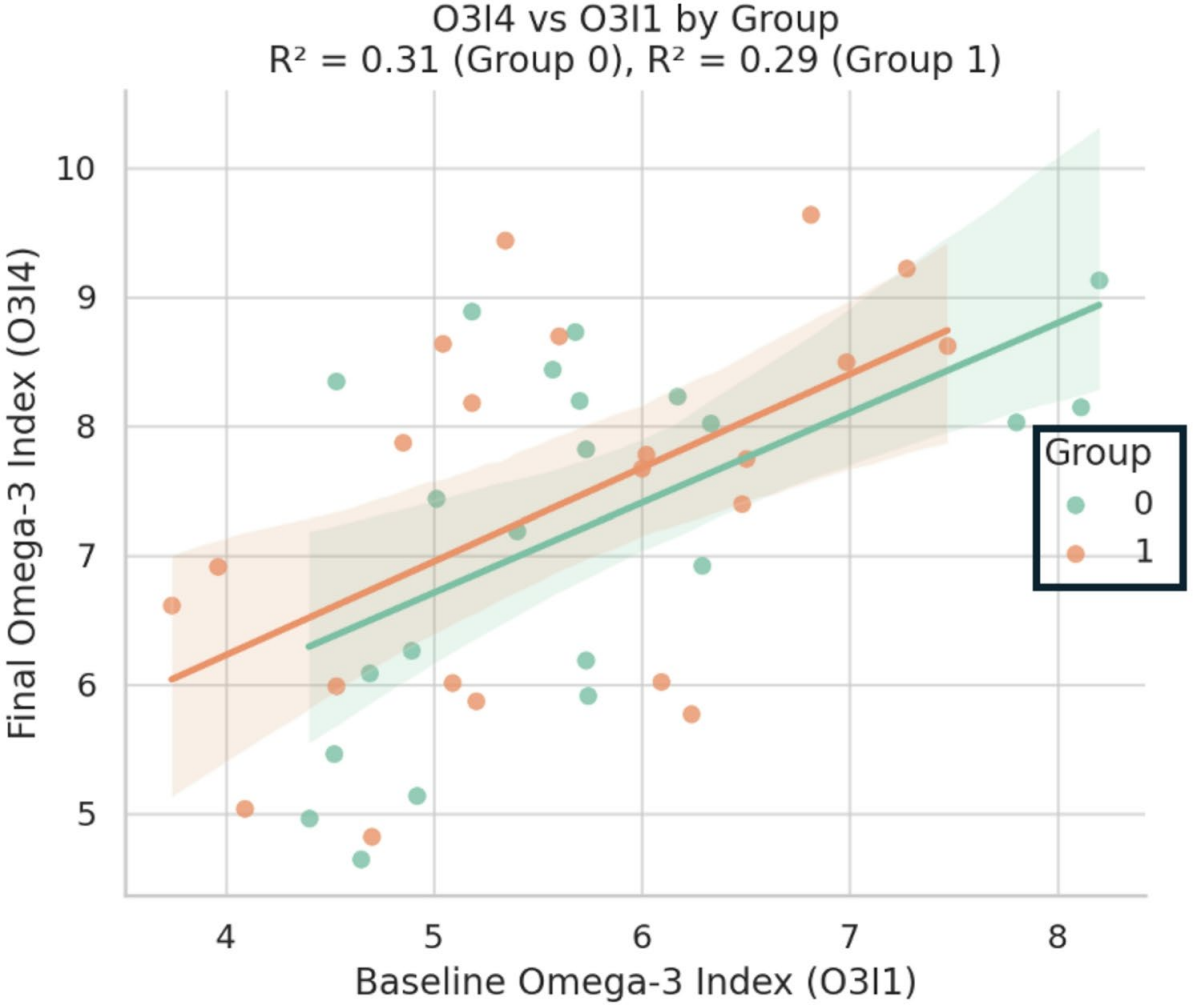
Correlation between baseline and final omega-3 Index by group

Similarly, both groups demonstrated a strong linear relationship between baseline and final O31 values (Fig. 8). The intervention group had a slightly higher intercept, indicating that for a given O3I1 value, participants receiving the phospholipid-bound formulation achieved greater O3I4 enrichment. The explanatory power was similar (R² = 0.31 in the control group, R² = 0.29 in the intervention group), reinforcing the improved bioavailability associated with phospholipid-based delivery systems.

These subgroup-specific analyses support a favorable trend in biomarker responses for the PL omega-3 formulation, particularly in individuals with elevated baseline TG or lower initial O3I.

Scatter plot with regression lines showing the relationship between baseline (O3I1) and final (O3I4) omega-3 Index values. The control group (green line = 0) showed a slightly higher coefficient of determination (R² = 0.31) compared to the intervention group receiving phospholipid-bound omega-3 (orange line = 1; R² = 0.29). Both groups exhibited strong linear associations, indicating a consistent response pattern in omega-3 tissue incorporation relative to baseline levels.

#### Secondary outcomes

Secondary outcomes showed no significant differences in glucose, insulin, HOMA-IR, TyG index, TG/HDL ratio, HDL-C, LDL-C, non-HDL-C, TC, BMI, WC, HC, WHR, or blood pressure. Inflammatory markers (IL-6 and hsCRP) exhibited high variability and no consistent changes.

#### Glucose metabolism and insulin resistance

After 12 weeks of intervention, no statistically significant changes were observed in glucose metabolism or insulin resistance markers within or between groups. However, several trends may have clinical relevance and deserve further investigation in larger cohorts.

In the PL omega-3 formulation group, fasting glucose decreased modestly (mean Δ = − 2.1 mg/dL), whereas the control group exhibited a slight increase (+ 4.3 mg/dL), though this inter-group difference did not reach statistical significance (*p* = 0.464).

HOMA-IR, a surrogate index of hepatic insulin resistance, showed small, non-significant reductions in both groups (PL omega-3 formulation: − 0.10; Control: − 0.25), with wide inter-individual variability limiting the power to detect significant effects.

The TyG index, an emerging marker of insulin resistance relevant to metabolic syndrome, demonstrated a favorable downward trend in the PL omega-3 formulation group (mean Δ = − 0.05), while increasing slightly in the control group (+ 0.03). Despite these directional differences, no statistically significant changes were observed (*p* > 0.1).

Similarly, the TG/HDL-C ratio, another surrogate marker of insulin resistance and cardiometabolic risk, remained stable in both groups (PL omega-3 formulation: − 0.05; control: − 0.02), indicating limited short-term responsiveness to the intervention.

These findings suggest that while PL omega-3 formulation did not significantly impact glycemic or insulin resistance indices over the study period, there were subtle trends indicating potential metabolic benefits that warrant exploration in larger, adequately powered trials.

#### Lipid profile analysis

Analysis of the lipid profile using non-parametric methods showed a significant increase in LDL-C in the control group over the 12 weeks (*p* = 0.008, Wilcoxon signed-rank test), whereas no significant change was observed in the PL omega-3 group (*p* = 0.181). However, the between-group comparison of LDL-C changes did not reach statistical significance (*p* = 0.520, Mann–Whitney U test).

HDL-C levels remained stable in both groups, with no significant intra-group differences (control: *p* = 0.577; intervention: *p* = 0.627) and no significant between-group difference (*p* = 0.973).

Regarding non-HDL-C, neither group exhibited significant changes over time (control: *p* = 0.750; intervention: *p* = 0.924), and the inter-group comparison was also non-significant (*p* = 0.550).

These findings suggest that the PL omega-3 formulation did not significantly affect LDL, HDL, or non-HDL cholesterol levels compared to standard omega-3. Nonetheless, it may have attenuated the LDL-C increase observed in the control group.

#### Inflammatory markers

Systemic inflammation was assessed using IL-6 and hsCRP. Due to notable inter-individual variability, outlier exclusion was performed using the 1.5×IQR rule before statistical analysis. The IQR was calculated as the difference between the third quartile (Q3) and the first quartile (Q1), and values outside the range of Q1 − 1.5×IQR to Q3 + 1.5×IQR were excluded. This method is robust to skewed data and minimizes the influence of extreme responses, thereby improving the reliability of group comparisons.

##### IL-6 (pg/mL)

IL-6 concentrations remained stable in both study groups after 12 weeks. In the PL omega-3 group, levels changed from 2.44 ± 0.83 to 2.51 ± 1.14 pg/mL (*p* = 0.843). In the control group, values increased slightly from 2.44 ± 0.65 to 2.58 ± 0.98 pg/mL (*p* = 0.589), though not significantly. The between-group comparison of IL-6 changes was also non-significant (*p* = 0.851). All values remained within standard reference ranges.

##### HsCRP (mg/L)

A similar pattern was observed for hsCRP. In the intervention group, concentrations rose modestly from 2.95 ± 2.98 to 3.62 ± 3.89 mg/L (*p* = 0.388). In the control group, values increased from 3.41 ± 3.87 to 4.27 ± 5.08 mg/L (*p* = 0.385). The inter-group difference in hsCRP changes was not statistically significant (*p* = 0.879).

These findings suggest that the intervention did not induce significant changes in systemic inflammatory markers over the 12-week study period.

#### Anthropometric outcomes

Changes in body mass and regional fat distribution were assessed via BMI, WC, HC, and WHR. At baseline, both groups were classified on average within the overweight to obesity class I range.

After 12 weeks of intervention, no significant change in BMI was observed in either group (Control: +0.44 kg/m², *p* = 0.573; PL omega-3 formulation: − 0.32 kg/m², *p* = 0.686). WC showed a non-significant reduction in the PL omega-3 formulation group (from 101.1 cm to 99.6 cm, *p* = 0.159), while increasing in the control group (from 100.9 cm to 106.4 cm, *p* = 0.237).

Notably, HC rose significantly in the control group (from 103.2 cm to 106.8 cm, *p* = 0.003), while remaining unchanged in the PL omega-3 formulation (*p* = 0.801). Consequently, WHR remained stable in the control group (from 0.979 to 0.999, *p* = 0.670), but exhibited a favorable, though not statistically significant, reduction in the PL omega-3 formulation group (from 0.962 to 0.946, *p* = 0.204).

These trends may indicate a modest shift toward a more favorable fat distribution in the PL omega-3 formulation group, potentially reflecting subtle metabolic effects not captured by weight alone. The lack of statistical significance may be attributable to sample size limitations or biological variability.

#### Blood pressure outcomes

Blood pressure was recorded at baseline and after 12 weeks, including systolic blood pressure (SBP), diastolic blood pressure (DBP), and mean arterial pressure (MAP). At baseline, both groups exhibited values consistent with prehypertension or stage 1 hypertension.

After the intervention, SBP decreased by 5.95 mmHg in the PL omega-3 formulation group, compared with a 1.95 mmHg increase in the control group; however, these changes were not statistically significant (*p* = 0.186 and 0.306, respectively). Similarly, DBP decreased by 4.41 mmHg in the PL omega-3 formulation group and increased by 2.55 mmHg in the control group (*p* = 0.093 and 0.245, respectively).

Importantly, MAP decreased significantly in both groups, with a more pronounced reduction in the PL omega-3 formulation group (from 102.7 to 95.9 mmHg, *p* < 0.001) compared to the control group (from 95.7 to 91.6 mmHg, *p* < 0.001).

These findings suggest that both interventions may contribute to lowering arterial tone over time, but the greater reductions observed in the PL omega-3 formulation group support a potentially enhanced antihypertensive effect. This could be mediated through improved endothelial function, anti-inflammatory activity, or modulation of sympathetic tone, consistent with prior studies on PL omega-3 formulations [5–11].

#### Lifestyle factors: diet and physical activity

Lifestyle habits, including dietary adherence and exercise frequency, were evaluated to assess potential confounding or synergistic influences on metabolic outcomes.

#### Dietary habits

At baseline, 27.3% of participants in the PL omega-3 formulation group and 18.2% in the control group reported following a structured diet. Most participants (72.7% in the PL omega-3 formulation and 81.8% in the control) did not adhere to any specific dietary regimen. Among those reporting dietary adherence, phospholipid-bound omega-3 participants more frequently followed the Mediterranean (33.3%) or DASH (16.7%) diets, whereas the control group favored hypoglycemic (25%) or low-sodium (25%) diets. This distribution reflects greater dietary diversity within the phospholipid-bound omega-3 group.

#### Exercise frequency

Physical activity was assessed based on self-reported frequency at baseline and week 12. Most participants in both groups maintained their baseline activity levels throughout the intervention (PL omega-3 formulation: 54.5%; control: 50%). Among PL omega-3 formulation participants, 18.2% reported an increase in exercise frequency by two categorical levels, compared to only 4.5% in the control group. Interestingly, an equivalent proportion (18.2%) of PL omega-3 formulation participants also reported a two-level decrease in activity.

These opposing shifts resulted in a net neutral trend, reflecting high individual variability and the absence of a consistent directional change in exercise behavior. Although the PL omega-3 formulation group exhibited a slightly greater inclination toward lifestyle modifications, the small sample size and self-reported nature of these measures preclude definitive conclusions. Future studies should include objective assessments and standardized counseling to better characterize the interplay between behavioral changes and metabolic outcomes.

#### Safety analysis

All hematological, hepatic, and renal safety parameters remained within clinically acceptable ranges throughout the 12-week intervention in both treatment groups. The only statistically significant laboratory change observed was an increase in PTT in the phospholipid-bound omega-3 formulation group (*p* = 0.0345). This change was not associated with any clinical symptoms or adverse bleeding events, suggesting a probable subclinical anticoagulant effect of the PL omega-3 formulation that warrants further investigation.

#### Safety and adverse events

**Fig. 9:**
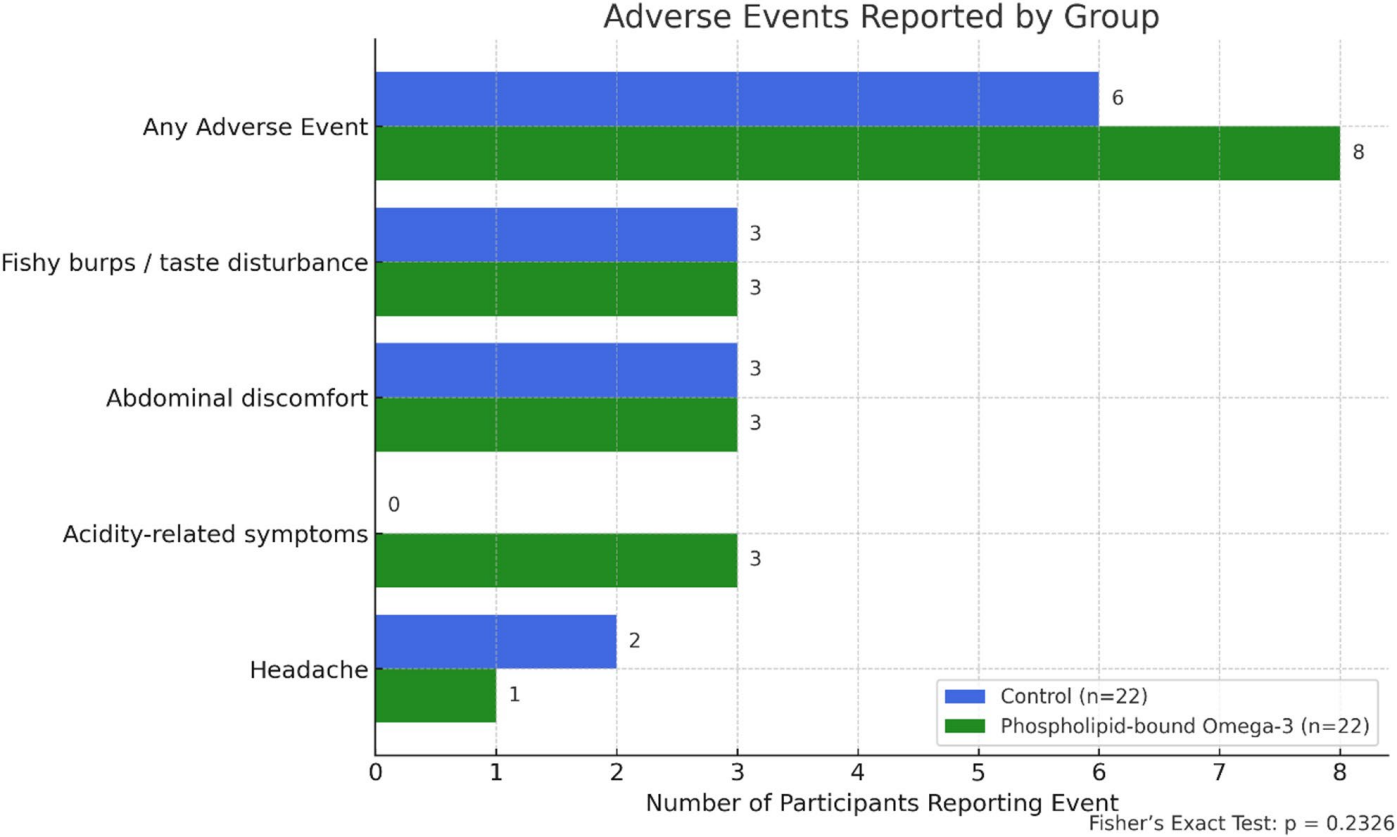
Adverse events reported by the treatment group

Adverse events were reported in 8 participants (36.4%) in the PL omega-3 group and 6 participants (27.3%) in the control group (Fig. 9). All events were mild, transient, and self-limited, and no participant required treatment discontinuation, medical intervention, or dose modification. No serious adverse events occurred in either group.

The most frequently reported symptoms were eructations with a fishy aftertaste, taste disturbances, and abdominal discomfort, all of which occurred at similar frequencies in both groups. Acidity-related symptoms (e.g., gastritis or reflux) were observed only in the PL omega-3 group (3 cases), although the difference was not statistically significant (Fisher’s exact test, *p* = 0.2326). Headache occurred slightly more often in the control group, likewise without a significant difference.

No individual adverse event differed significantly between groups. All symptoms typically resolved spontaneously within a few days and were consistent with the known tolerability profile of short-term omega-3 supplementation. Overall, both formulations were well tolerated, and adherence remained high throughout the study, with a safety profile comparable to previous omega-3 supplementation trials.

Bar chart summarizing the number of participants in each group who reported adverse events during the 12-week trial. The most frequently reported events were taste disturbances and abdominal discomfort, which occurred equally in both groups (*n* = 3 each). Acidity-related symptoms were only reported in the intervention group (phospholipid-bound omega-3), whereas headaches were more frequent in the control group. Overall adverse event rates did not differ significantly between groups (Fisher’s exact test, *p* = 0.2326; *n* = 22 per group).

## Discussion

This pilot trial aimed to evaluate whether a PL omega-3 formulation could enhance TG management in patients with moderate hypertriglyceridemia. Although group-level reductions in serum TG were modest and did not reach statistical significance, the formulation achieved a significantly higher proportion of responders at clinically meaningful thresholds. These findings underscore the potential clinical utility of optimized omega-3 delivery systems, particularly for patients with residual lipid-related risk.

After adjusting for total daily EPA + DHA intake (825 mg/day in the phospholipid-bound omega-3 group vs. 903 mg/day in the control group), the phospholipid formulation exhibited superior dose efficiency. The dose-normalized O3I response was calculated as 0.0422% per mg in the phospholipid group versus 0.0311% per mg in controls, corresponding to a Relative Efficiency Index (REI) of 1.34, indicating 34% higher biological incorporation per milligram of active compound. This improved efficiency is likely attributed to the inclusion of functional phospholipids such as phosphatidylcholine (PC), lysophosphatidylcholine (LPC), phosphatidylethanolamine (PE), and phosphatidylinositol (PI), which are known to enhance gastrointestinal stability and facilitate membrane-based uptake of long-chain polyunsaturated fatty acids. Notably, Schuchardt et al. demonstrated in a randomized crossover trial that krill oil—rich in phospholipid-bound EPA and DHA—achieves superior incorporation into plasma phospholipids compared to triglyceride or ethyl ester formulations [14].

Phospholipids are fundamental components of cell membranes and serve as potent bioactive vectors in both nutraceutical and pharmaceutical applications. Their amphipathic structure supports micelle and liposome formation, thereby increasing solubility, dispersion, and intestinal absorption of hydrophobic bioactives such as EPA and DHA. Beyond their structural roles, phospholipids exhibit diverse biological effects, including modulation of pro-inflammatory cytokines (e.g., TNF-α, IL-6), oxidative stress attenuation through membrane stabilization, and regulation of metabolic and signaling pathways such as PPARs and NF-κB [15].

Multiple mechanisms may account for the superior performance of phospholipid-bound omega-3s compared to standard triglyceride-based formulations. Ramprasath et al. reported that krill oil supplementation leads to significantly greater plasma enrichment of EPA and DHA than fish oil at equimolar dosages [16]. In preclinical studies, phospholipid-bound DHA demonstrated enhanced incorporation into hepatic and myocardial tissues relative to non-polar forms [17]. Furthermore, PL omega-3s appear to activate nuclear receptors such as PPARα and HNF4α, which are involved in the transcriptional regulation of lipid metabolism and inflammatory pathways [18].

Although no statistically significant changes in CRP or IL-6 were observed in our study, the modest yet consistent increase in partial thromboplastin time (PTT) within the PL omega-3 group may reflect mild anticoagulant effects. This observation is supported by previous findings showing that omega-3 fatty acids inhibit thromboxane A2 production and reduce platelet aggregation, effects potentially amplified by phospholipid-mediated transport [19, 20]. The clinical relevance of these hemostatic shifts, while remaining within reference ranges, warrants further exploration in adequately powered trials.

The increase in O3I in both arms of the study confirms satisfactory compliance; however, the greater absolute rise in the PL omega-3 groups (+ 1.88 vs.+1.45) reinforces the hypothesis of superior tissue delivery and retention. Additionally, the proportion of patients achieving triglyceride thresholds below 166 and 156 mg/dL was significantly higher in the intervention group, supporting its role in personalized lipid-lowering strategies, especially among statin-intolerant or high-residual-risk populations.

The clinical relevance of the 166 mg/dL triglyceride threshold is supported by a large-scale epidemiological study involving over 1.8 million Korean adults, which demonstrated a 45% increased risk of incident cardiovascular events in individuals exceeding this level, independent of LDL-C, obesity, diabetes, and hypertension [21]. Thus, our adoption of this cutoff is grounded in both statistical distribution and robust prognostic validation, facilitating risk stratification in real-world settings.

Lu et al. previously demonstrated that conventional fish oil reduces TG levels in patients with type 2 diabetes. However, the magnitude of response varied substantially across individuals, likely influenced by lipidomic profiles, baseline omega-3 status, and gut microbiota composition [22]. These findings reinforce the rationale for precision nutrition approaches and suggest that PL omega-3s, such as *Ruby-O*^®^ Balance, may overcome variability through improved bioavailability and cellular uptake.

Comparative studies of krill oil provide additional support. Berge et al. found that krill oil reduced triglycerides without raising LDL-C—a common concern with fish oil [23]—while Ulven et al. reported substantial TG reductions at lower doses, attributed to improved gastrointestinal absorption and membrane incorporation [24]. A network meta-analysis by Derosa et al. concluded that krill oil demonstrated comparable or superior lipid-lowering efficacy with fewer gastrointestinal side effects than fish oil [25].

Although a numerically higher frequency of mild gastrointestinal symptoms was observed in the PL group, these events were non-serious, self-limited, and consistent with the known tolerability profile of omega-3 formulations. No clinically meaningful safety imbalances were identified, and the small sample size precludes definitive interpretation of this difference. These findings are consistent with the favorable safety profile observed in our cohort, as all adverse events were mild and self-limiting.

The potential anticoagulant properties of omega-3 fatty acids have been described in both preclinical and clinical settings. Phang et al. demonstrated reductions in platelet aggregation and thromboxane production following supplementation [19], while von Schacky provided a broader context on the cardiovascular relevance of omega-3 status [20]. Although most clinical trials have not shown consistent changes in prothrombin time (PT) or partial thromboplastin time (aPTT) [26], animal models support antithrombotic effects through inhibition of platelet function and thrombus formation [12]. Jump et al. further emphasized the role of PPAR-α activation in modulating inflammatory and coagulation pathways [18].

The statistically significant but clinically benign increase in PTT observed in the PL omega-3 group may represent a subclinical hemostatic effect mediated by enhanced cellular delivery of omega-3s. Future randomized trials including coagulation-specific endpoints are needed to evaluate this hypothesis in the context of cardiovascular risk reduction.

Inter-individual variability in TG response to omega-3 therapy is well documented. As reviewed by Rundblad et al. [27], genetic polymorphisms—particularly in genes involved in lipid metabolism and transcriptional regulation (e.g., PPARA, APOA5, FADS1/FADS2, LPL, CETP, and SREBP)—modulate responsiveness to omega-3 supplementation. For example, the L162V polymorphism in PPARA (rs1800206) has been associated with increased sensitivity to EPA, resulting in greater TG reductions [28]. In contrast, the − 1131T > C variant in APOA5 (rs662799) impairs TG clearance and is linked to attenuated responsiveness to standard omega-3 therapy [29]. Likewise, the rs174537 SNP in FADS1 reduces endogenous synthesis of EPA/DHA, limiting omega-3 incorporation into tissues unless preformed fatty acids are provided [30].

Epigenetic factors, non-coding RNAs, and gut microbiota also contribute to phenotypic variability [27]. In this study, we evaluated a novel PL omega-3 delivery system incorporating EPA and DHA into a matrix of polar phospholipids (see Supplementary Material) designed to enhance membrane integration and metabolic efficacy. This formulation may overcome certain genetic limitations by promoting superior absorption and cellular delivery. Indeed, phosphatidylcholine and phosphatidylethanolamine, present in our PL omega-3, facilitate bypass of rate-limiting steps in lipid transport and enzymatic desaturation [31].

Greater benefits have been observed in individuals with higher baseline TG and lower habitual omega-3 intake, highlighting the clinical potential of phospholipid-bound omega-3 formulations in personalized lipid therapy. These findings suggest that future clinical trials could benefit from including pharmacogenetic stratification to improve therapeutic outcomes [27, 28]. As emphasized by Ordovás and Shen, the lipid-modifying effects of polyunsaturated fatty acids, including omega-3s, are influenced by genetic variation, supporting the rationale for personalized lipid therapy based on gene–nutrient interactions [32]. Additionally, von Schacky has championed the HS-omega-3 Index as the most reliable biomarker for long-term omega-3 status, aiding both clinical monitoring and research standardization [33].

Unlike dietary assessments or plasma measures, which are limited by short-term variability and recall bias, the O3I reflects red blood cell membrane incorporation over approximately 120 days, with lower intra-individual variability (~ 4.1%). Values between 8% and 11% are associated with reduced cardiovascular morbidity and mortality. Importantly, this biomarker supports a personalized, physiology-based approach to dosing, as clinical outcomes in major trials such as REDUCE-IT [34], JELIS [35], ASCEND [36], and STRENGTH [37] correlate more strongly with achieved index levels than with fixed doses.

Despite its strengths, the O3I has limitations. It does not distinguish between EPA and DHA contributions and may not fully capture tissue-specific activity. Genetic variability in desaturase enzymes (e.g., FADS), PPAR signaling, and membrane transport proteins may further affect baseline values and treatment response. These factors should be considered in future trial designs.

Recent advances underscore the dual relevance of phospholipids and omega-3 fatty acids in cardiovascular health. Phospholipids such as phosphatidylserine (PS) and sphingosine-1-phosphate (S1P) modulate post-infarction myocardial repair by regulating inflammatory resolution, fibrosis, angiogenesis, and mitochondrial function [38]. Meanwhile, persistent hypertriglyceridemia—often exacerbated by therapeutic inertia—remains a key contributor to residual cardiovascular risk [38]. In this context, PL omega-3 demonstrated improved triglyceride response rates and enhanced O3I incorporation, supporting its utility in optimizing lipid management for high-risk patients. A large, randomized trial showed that high-dose omega-3 (4 g/day EPA/DHA) significantly lowered triglycerides and non–HDL-C in statin-treated individuals with residual hypertriglyceridemia [39]. Phospholipid-bound formulations may enhance these benefits through superior bioavailability and tissue distribution.

### Limitations and rationale for further research

This study has several limitations that should be acknowledged. First, the relatively small sample size limits the statistical power to detect subtle differences, particularly in secondary outcomes. Second, the short intervention period may not fully capture long-term physiological adaptations or the sustainability of observed effects. Third, several endpoints—such as biomarker changes and indices of lipid metabolism—were exploratory in nature. These considerations reinforce the preliminary character of this trial and highlight the need for cautious interpretation of between-group comparisons.

Despite these constraints, the magnitude and direction of changes in clinically relevant outcomes, such as responder rates and O3I improvements, suggest a potentially meaningful biological effect. Collectively, these findings provide a strong rationale for conducting larger, longer-term randomized controlled trials to validate these preliminary observations and further elucidate the mechanisms underlying the potential therapeutic advantages of phospholipid-bound omega-3 formulations.

## Conclusion

In this pilot study, phospholipid-bound omega-3 supplementation did not produce statistically significant differences in TG reduction compared with standard omega-3. However, a significantly higher proportion of responders and a trend toward greater O3I improvement at a lower dose suggest potential biological advantages related to enhanced absorption and membrane incorporation. All adverse events were mild and self-limited.

These findings should be interpreted as preliminary and hypothesis-generating, supporting the rationale for larger, adequately powered randomized trials to further assess the clinical relevance of phospholipid-bound omega-3 formulations in patients with mild to moderate hypertriglyceridemia.

## Supplementary Information

Supplementary Material 1.

Supplementary Material 2.

Supplementary Material 3.

Supplementary Material 4.

## Data Availability

The study protocol and statistical analysis plan were registered on *ClinicalTrials.gov* (NCT06749028) and are available upon reasonable request from the corresponding author. De-identified participant data and statistical analysis code are also available upon reasonable request for academic and research purposes.

## Abbreviations

TG: Triglycerides
O3I: Omega-3 Index
EPA: Eicosapentaenoic Acid
DHA: Docosahexaenoic Acid
HDL-C: High-Density Lipoprotein Cholesterol
LDL-C: Low-Density Lipoprotein Cholesterol
hsCRP: High-Sensitivity C-Reactive Protein
IL-6: Interleukin-6
BMI: Body Mass Index
WC: Waist Circumference
HC: Hip Circumference
WHR: Waist-to-Hip Ratio
MAP: Mean Arterial Pressure
TC: Total Cholesterol
HOMA-IR: Homeostasis Model Assessment of Insulin Resistance
TyG index: Triglyceride-Glucose Index
PT: Prothrombin Time
PTT: Partial Thromboplastin Time
INR: International Normalized Ratio
BUN: Blood Urea Nitrogen
GFR: Glomerular Filtration Rate
AST: Aspartate Aminotransferase
ALT: Alanine Aminotransferase
ALP: Alkaline Phosphatase
GGT: Gamma-Glutamyl Transferase
