# Supplementary figures and images for: Efficacy of phospholipid-bound omega-3 versus standard omega-3 in patients with hypertriglyceridemia: a randomized clinical trial
Source: BMC Complement Med Ther. 2026 Jan 10;26:48. doi: 10.1186/s12906-026-05245-1 (PMC12882287; doi:10.1186/s12906-026-05245-1)

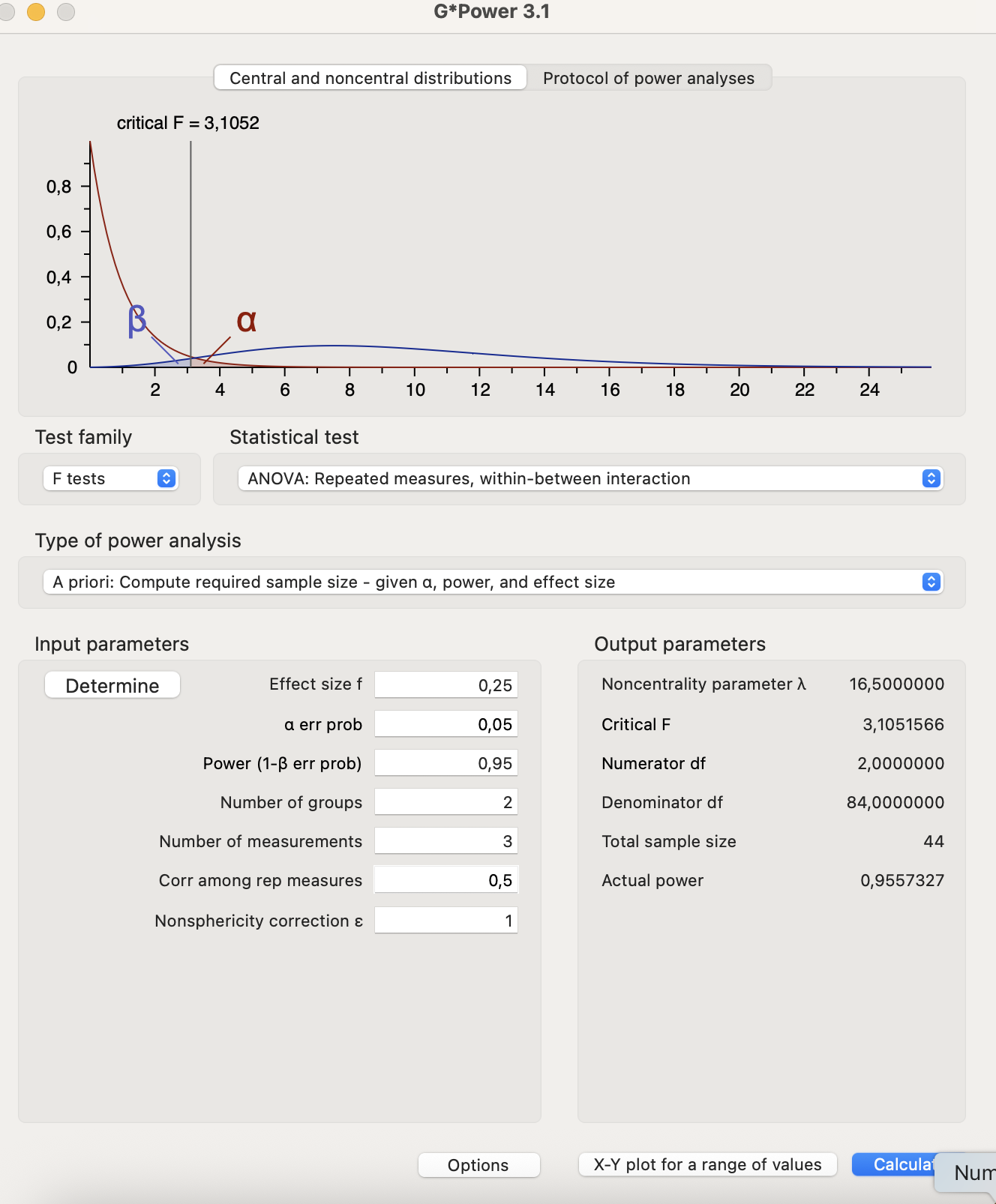

Supplement: Supplementary file 1 — Supplementary Material 1. [file 12906_2026_5245_MOESM1_ESM.docx]
