# Supplementary material for: Efficacy of phospholipid-bound omega-3 versus standard omega-3 in patients with hypertriglyceridemia: a randomized clinical trial
Source: BMC Complement Med Ther. 2026 Jan 10;26:48. doi: 10.1186/s12906-026-05245-1 (PMC12882287; doi:10.1186/s12906-026-05245-1)
Supplement: Supplementary file 2 — Supplementary Material 2. [file 12906_2026_5245_MOESM2_ESM.docx]

**
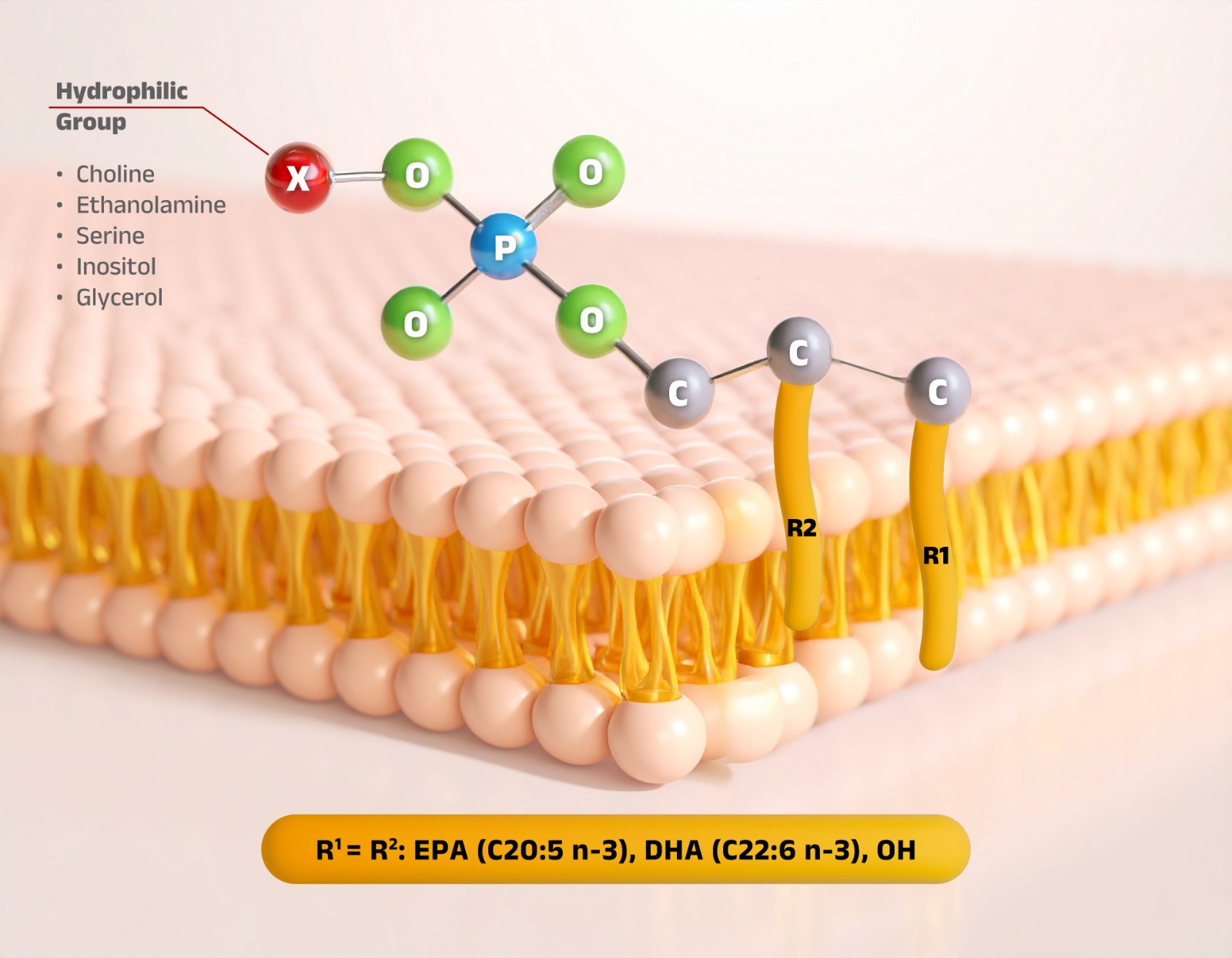
**

**Appendix product information**

Ruby-O® Balance (phospholipid-bound Omega-3 formulation) has been developed as an advanced omega-3 formulation that combines EPA and DHA (non-polar lipids) with a matrix of phospholipids (polar lipids) covalently bound to omega-3 fatty acids (EPA+DHA). This matrix is primarily composed of phosphatidylcholine (PC), lysophosphatidylcholine (LPC), phosphatidylinositol (PI), phosphatidylethanolamine (PE), and other structural phospholipids that are naturally present in cell membranes.

This molecular architecture is designed to enhance the efficiency of omega-3 absorption and metabolic utilization by promoting their direct incorporation into cellular membranes, reducing susceptibility to oxidation, and facilitating a more efficient and sustained tissue distribution.
