# Supplementary material for: Efficacy of phospholipid-bound omega-3 versus standard omega-3 in patients with hypertriglyceridemia: a randomized clinical trial
Source: BMC Complement Med Ther. 2026 Jan 10;26:48. doi: 10.1186/s12906-026-05245-1 (PMC12882287; doi:10.1186/s12906-026-05245-1)
Supplement: Supplementary file 4 — Supplementary Material 4. [file 12906_2026_5245_MOESM4_ESM.pdf]

Supplementary Material 1. Analytical characterization and compositional profile of the investigational phospholipid-bound omega-3 (Ruby-O® Balance) and standard omega-3 fish oil (TG 18/12, Essentiomega™).

Both the Omega-3 fish oil product (TG 18/12, Essentiomega™) and the phospholipid-bound Omega-3 formulation (Ruby-O® Balance, patented technology[1]) were manufactured by C.I. Naturmega S.A. (Barranquilla, Colombia). Sodium hydroxide (0.1 M), n-heptane, dichloromethane, ethyl acetate, acetone, acetic acid, methanol, iso-octane, boron trifluoride, sodium chloride, triphenylphosphine, cesium carbonate, deuterated chloroform, deuterated methanol, EDTA, deuterated water, and standards of PC, sn1-LPC, and PE were obtained from MERCK (Germany). Dichloromethane, acetone, acetic acid, and chloroform were also purchased from Sigma-Aldrich, Inc. (Darmstadt, Germany). All solvents and reagents were of analytical grade. GC-FID and HPLC standards were obtained from Sigma–Aldrich (Castle Hill, Australia). Silica gel was purchased from SiliCycle Inc. (Quebec, Canada).

NMR spectra were obtained using a Bruker Advance 400 MHz spectrometer. All chemical shifts observed in the NMR experiments were reported in parts per million (ppm) relative to TMS, with a downfield shift. The calibration values used were 7.26 ppm for ^{1}H NMR in $CDCl_3$ and 77.0 ppm for ^{13}C NMR. For the ^{31}P NMR analysis, a single sample was analyzed under consistent conditions, including a temperature of 25°C, 600 scans, a relaxation delay of 3.5 s, a sweep width of 6,067 Hz, 65,536 data points, and an excitation pulse of 90°. Proton decoupling was applied during the analysis.

1.2. Extraction of Omega-3–Bound Phospholipids

To isolate the phospholipids bound to omega-3 fatty acids, a column chromatography separation was performed using silica gel as the stationary phase. Elution was carried out with a solvent system composed of chloroform, acetone, methanol, and glacial acetic acid (60:1:1:1, v/v). The collected fractions were subsequently verified by thin-layer chromatography (TLC) to confirm the proper separation of the phospholipid components [2].

1.3. Fatty Acid Profile by GC-FID for Omega-3 Determination

The fatty acid profile analysis was performed following the methodology reported by Qing Shen [3], with minor modifications. Specifically, 30 mg of the phospholipids previously isolated by column chromatography were mixed with 2 mL of sodium methoxide solution (NaOH in methanol, 0.5 M) and heated at 80 °C for 30 minutes. After cooling to room temperature, 2 mL of boron trifluoride in methanol (BF_3-MeOH) were added, and the mixture was heated again at 65 °C for 15 minutes.

The resulting product was cooled, extracted with 2 mL of iso-octane, and washed with 5 mL of saturated NaCl solution. The upper organic phase was recovered, dried over anhydrous sodium sulfate, and filtered through a 0.22 µm membrane prior to chromatographic analysis.

Fatty acid methyl esters (FAMEs) were separated using an HP-88 capillary column (30 m × 0.25 mm i.d., 0.20 µm film thickness, 100% cyanopropyl polysiloxane; Agilent Technologies, DE, USA) installed on an Agilent 7890A GC system equipped with a flame ionization detector (FID). One microliter (1 µL) of each sample was injected in split

mode (40:1), using high-purity nitrogen as the carrier gas at a flow rate of 0.65 mL/min. The FID temperature was set to 250 °C. The oven temperature program consisted of an initial hold at 50 °C for 2 minutes, followed by an increase to 220 °C at a rate of 4 °C/min. The column was subsequently cooled for 15 minutes. All samples were analyzed in triplicate.Additionally, the quantification of total omega-3 content in the products was performed following GOED guidelines and the methodology specified in USP monograph 401 for the different chemical forms of omega-3 fatty acids [4].

1.4 Sample preparation for NMR analysis.

To prepare phospholipid-bound Omega-3 formulation, we followed the procedure outlined by Yulia B. Monakhova [5]. In a centrifuge tube, we combined 210-280 mg of the sample with 1.5 mL of $CDCl_3$, 25-28 mg of TPP(Triphenylphosphine) as an internal standard, 3 mL of CD_3OD, and 3 mL of a Cs-EDTA stock solution. Subsequently, the solution was vigorously mixed for 5 minutes and then subjected to centrifugation at 1,900 rpm for 10 minutes using a tabletop centrifuge (Beckman Coulter, Inc., Fullerton, CA, USA). The lower chloroform layer was carefully transferred to a 5 mm NMR tube for subsequent analysis. The preparation of the Cs-EDTA stock solution involved weighing approximately 2.9 g of EDTA and 6 g of Cs_2CO_3, which were dissolved in 50 mL of D_2O. The pH was then adjusted to 7.5 as part of the process.

The selection of TPP as an internal standard for this analysis is grounded in its complete extraction in the organic layer, its acceptable T1 time, its non-reactivity with

phospholipids, and the presence of a phosphorus atom in its structure [6]. Regarding the assignment of signals in the ^{31}P NMR spectrum, commercial standards were individually run (Please refer to the appendices for further details), and for signals of PL where standards were unavailable, assignment was based on previously reported literature. It's worth noting that the ^{31}P NMR analysis of PL provides high selectivity, allowing differentiation of the most common PL classes without the need for reference standards [7].

2. Analytical results obtained from fatty acid profile using GC-FID for the determination of omega-3

Table 1 presents the detailed lipid composition (g/100 g) of the phospholipid fraction previously isolated by column chromatography from the Phospholipid-Bound Omega-3 product, along with the composition of the Omega-3 Fish Oil product (TG 18/12). All analyses were performed in triplicate to ensure reproducibility. As shown, the Phospholipid-Bound Omega-3 product exhibits a total concentration of phospholipid-bound EPA+DHA of 38.37 g/100 g, consisting of 23.30 g/100 g of EPA and 15.07 g/100 g of DHA, confirming efficient incorporation of omega-3 fatty acids into the polar fraction.

It is important to note that, for the Fish Oil product, chromatographic separation was not required because this oil does not contain phospholipids. In contrast, the values reported for the Phospholipid-Bound Omega-3 product correspond exclusively to the isolated phospholipid fraction, which was subsequently analyzed according to the

procedure described previously.

Table 1, Fatty acid composition of the Omega-3 Fish Oil (TG 18/12) and phospholipid fraction obtained by column chromatography (g/100 g)

**Fatty acids**	**Product**	
	**Omega-3 Fish Oil TG 18/12 (g/100g)**	**Phospholipid-Bound Omega-3 (g/100g)**
C16:0	2.84 ± 0.21	6.26 ± 0.04
C18:0	5.70 ± 0.15	5.6 ± 0.06
C18:1 n-9	22.5 ± 0.31	8.89 ± 0.23
C18:1 n-7	2.19 ± 0.05	3.01 ± 0.06
C18:2 n-6	23.11 ± 0.04	20.89 ± 0.32
C18:3 n-3	0.56 ± 0.01	2.76 ± 0.03
C20:1 n-9	0.83 ± 0.21	1.5 ± 0.09
C20:5 n-3 (EPA)	18.88 ± 0.21	23.30 ± 0.16
C22:5 n-3	1.76 ± 0.14	3.18 ± 0.01
C22:6 n-3 (DHA)	13.88 ± 0.15	15.07 ± 0.17
Other fatty acids	7.75 ± 0.17	9.54 ± 0.43
Total	100	100

Complementarily, **Table 2** presents the total EPA+DHA concentration of each product, determined according to the GOED-recommended monograph

Table 2. Concentration (mg/g) of each product used in the study.

Fatty acids	Product	
	**Omega-3 Fish Oil TG 18/12 (g/100g)**	**Phospholipid-Bound Omega-3 (mg/g)**
C20:5 n-3 (EPA)	180	167
C18:0 (DHA)	121	108

2.1 NMR analysis of the Phospholipid-Bound Omega-3 product

To confirm the phospholipid composition of the Phospholipid-Bound Omega-3 product (g/100g), a ^{31}P NMR analysis was performed. Table 3 presents the quantification and distribution of the phospholipids identified in the sample. To ensure robust structural characterization, USP standards of individual phospholipids and defined mixtures were employed, allowing precise assignment of each observed signal.

As summarized in **Table 3**, twelve phospholipid species were identified, belonging to the PC, PI, sn1-LPC, sn2-LPC, LPS, PE, NAPE, DPG (cardiolipin), PG, LPE, PA, LPA (**see Figure 1**). Together, these species accounted for a mean total phospholipid content of 37.28 g/100 g, in addition to 3.52 g/100 g of the glycolipid MGDG (**See Figure 2**).

Table 3, Polar lipid composition (g/100 g) of Phospholipid-Bound Omega-3 product (Ruby-O Balance®)

Polar Lipids	NMR signal (ppm)	Concentration in g/100g	LB
Phosphatidylcholine (PC)	-0.75a	5.49 (± 0.65)	<0.1
Phosphatidylinositol (PI)	-0.54a	1.11 (± 0.17)	<0.1
Lyso-phosphatidylcholine, acylated at the glycerol 1-carbon (sn1-LPC)	-0.45a	11.86 (± 0.93)	<0.1
Lyso-phosphatidylcholine, acylated at the glycerol 2-carbon (sn2-LPC)	-0.08a	6.52 (± 0.35)	<0.1
Lyso-phosphatidylserine (LPS)	-0.06a	0.76 (± 0.18)	<0.1
PE (Phosphatidylethanolamine)	0.29a	2.07 (± 0.04)	<0.1
N-acyl phosphatidylethanolamine (NAPE)	0.51a	1.31 (± 0.38)	<0.1
DPG-Cardiolipin (Di phosphatidylglycerol)	0.68a	3.81 (± 0.78)	<0.1
Phosphatidylglycerol (PG)	0.82a	1.79 (± 0.26)	<0.1
Lyso-Phosphatidylethanolamine (LPE)	0.88a	0.51 (± 0.08)	<0.1
Phosphatidic acid (PA)	2.73a	0.75 (± 0.17)	<0.1
Lysophosphatidic acid LPA	4.19a	1.29 (± 0.36)	<0.1
Total phospholipids		37.28 (± 0.55)	<0.1
Monogalactosyl diacylglycerol (MGDG, glycolipid)	4.29b	3.52 (± 0.39)	<0.1
Total polar lipids		40.80 (± 0.39)	<0.1

a: ^{31}P NMR (the sample was analyzed in quintupled); b: ^{1}H NMR (the sample was analyzed in triplicate); LB: lower quantification limit.

Figure 1. ^{31}P NMR spectrum of the Phospholipid-Bound Omega-3 product

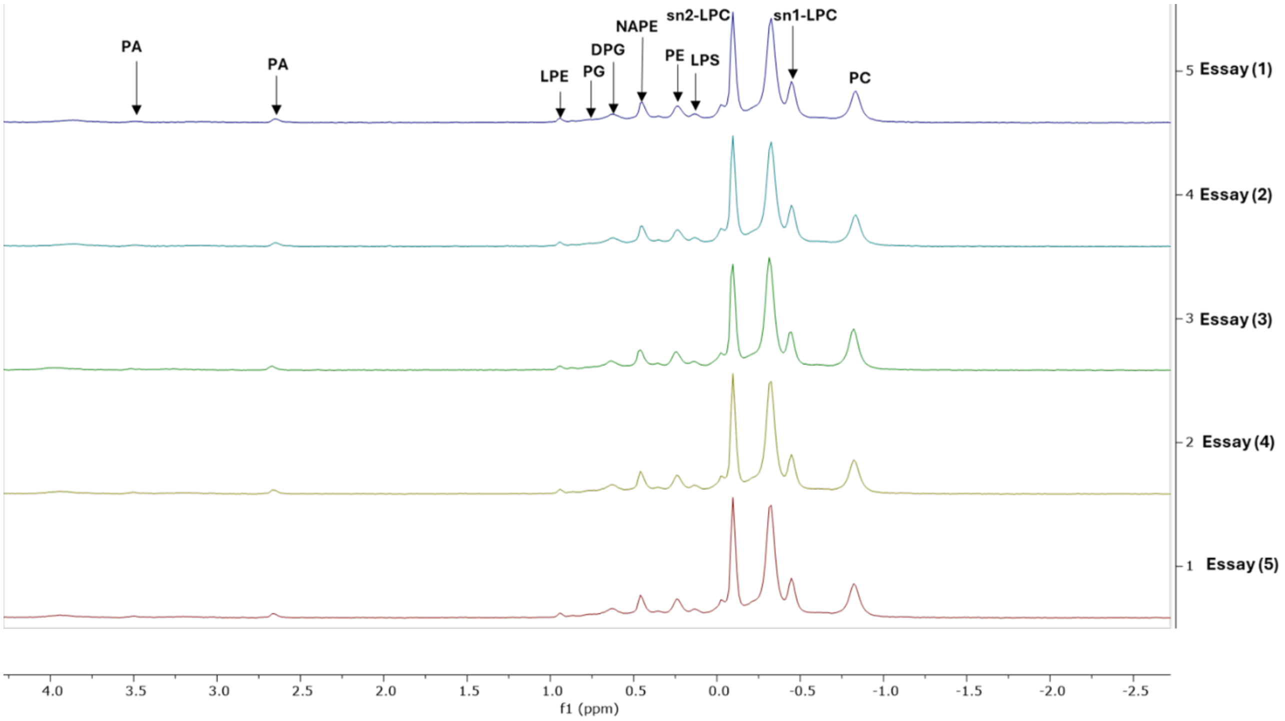

Figure 2. ^{1}H NMR spectrum of the Phospholipid-Bound Omega-3 product vs Standard MGDG

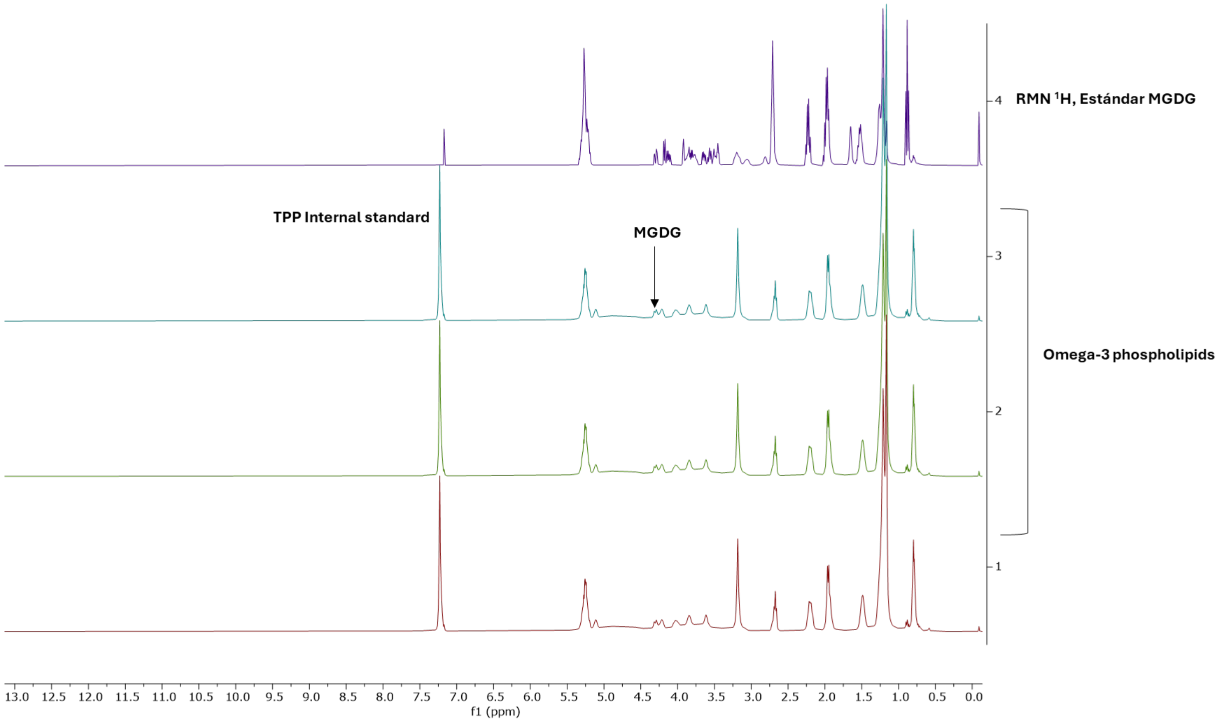

References

1. Gutierrez WM, Padilla AJG, Simanca ADJP, Bustillo TLY. Enzymatically synthesized omega-3 structured phospholipids. Technical report, Naturmega S.A.; Barranquilla, Colombia. 2023.

2. Skipski V, Peterson R, Barclay M. Quantitative analysis of phospholipids by thin-layer chromatography. Biochem J. 1964; 90:374–8. doi:10.1042/bj0900374.

3. Shen Q, Song G, Wang H, Zhang Y, Cui Y, Xie H, et al. Isolation and lipidomics characterization of fatty acids and phospholipids in shrimp waste through

GC/FID and HILIC-QTrap/MS. J Food Compos Anal. 2021; 95:103668. doi: 10.1016/j.jfca.2020.103668.

4. Global Organization for EPA and DHA Omega-3 (GOED). GOED Voluntary Monograph. Version 8.1. 2022 Jan 6. Available from: https://goedomega3.com/storage/app/media/Monograph/GOED%20Monograph%20-%202022%2001%2006%20-%20FINAL.pdf. Accessed 10 Dec 2025.

5. Monakhova YB, Diehl BWK. Automated multicomponent phospholipid analysis using 31P NMR spectroscopy: example of vegetable lecithin and krill oil. Anal Bioanal Chem. 2018; 410:7891–900. doi:10.1007/s00216-018-1408-9.

6. Diehl BWK, Monakhova YB. Method performance and validation for quantitative analysis by 1H and 31P NMR spectroscopy: applications to analytical standards and agricultural chemicals. Anal Chem. 1998; 70:4548–56. doi:10.1021/ac980573i.

7. Burri L, Hoem N, Monakhova YB, Diehl BWK. Fingerprinting krill oil by 31P, 1H and 13C NMR spectroscopies. J Am Oil Chem Soc. 2016; 93:1037–49. doi:10.1007/s11746-016-2836-3.
